# On the inaccuracies of dental radiometers

**DOI:** 10.1371/journal.pone.0245830

**Published:** 2021-01-29

**Authors:** Adrian C. Shortall, Mohammed A. Hadis, William M. Palin

**Affiliations:** College of Medical and Dental Sciences, Institute of Clinical Studies, University of Birmingham, Birmingham, United Kingdom; Eberhard-Karls-Universitat Tubingen Medizinische Fakultat, GERMANY

## Abstract

This study investigated the accuracy of sixteen models of commercial dental radiometers (DR) in measuring the output of thirty-eight LED light curing units (LCUs) compared with a 'gold standard' laboratory-grade spectrometer integrating-sphere (IS) assembly. Nineteen Type I (fiber-bundle light guide) and nineteen Type II (light source in head) LED LCUs were tested, some using different output modes and light guides, resulting in 61 test subsets per radiometer. Gold standard (GS) output measurements (n = 3) were taken using the IS and confirmed with two types of laboratory-grade power meter (PowerMax-Pro 150 HD and PM10-19C; Coherent). One DR (Bluephase Meter II, Ivoclar; BM II) allowed power (mW) as well as irradiance (mW/cm^2^) recordings. Irradiance readings (n = 3) for each DR/LCU were compared with the IS derived irradiance. Individual LCU irradiance values were normalized against IS data. The GS method yielded reproducible data with a 0.4% pooled coefficient of variation for the LCUs. Mean power values ranged from 0.19 W to 2.40 W. Overall power values for the laboratory-grade power meters were within 5% of GS values. Individual LCU/DR normalized irradiance values ranged from 7% to 535% of the GS; an order of magnitude greater than previous reports. BM II was the only radiometer to average within 20% of normalized pooled GS irradiance values, whereas other radiometers differed by up to 85%. Ten radiometers failed to provide any reading for 1 LCU. When tested with the PowerMax-Pro in high speed (20 kHz) mode, eight LCUs demonstrated pulsing outputs undetectable at the standard (10 Hz) data acquisition rate. Sufficient light exposure is critical for the successful curing of dental resin-based materials. Substantial discrepancies may occur between actual and estimated radiometric data using current DRs. More accurate DRs need to be developed. Manufacturers' accuracy claims for DRs should specify compatible LCUs and testing parameters.

## 1. Introduction

The dental light-curing unit (LCU) is an essential item of equipment in contemporary dental practice, and since the development of light-activated resin-based composites in the 1970s, over 1000 different types, makes, and models of LCUs have been marketed [1]. Quartz tungsten halogen (QTH) LCUs, once the mainstay of dental practise, have been replaced by light-emitting diode (LED) units because of significantly improved energy efficiency and claims of longer service life [2, 3]. Modern trends in LED-LCU manufacturer include; 1) “broad spectrum” units which incorporate multiple LEDs to deliver light in the visible-violet (~385–410 nm), blue (~450–515 nm) as well as intermediate ranges of violet-blue wavelengths (~410–450 nm) as exemplified by the LCUs tested in the current investigation (Tables 1 and, 2 and S1 Fig) and a trend towards ever higher claimed irradiance values under the premise that shorter exposure times at higher irradiance levels deliver sufficient energy for optimal polymerization [1–3].

**Table 1:** Type I LCU optical properties and dimensions.

Light curing unit	Manufacturer/ Distributor	Serial Number	Modes tested	External tip diameter* (mm)	Active diameter** (mm)	Active area** (mm^2^)	Wavelength Range (nm)	Violet/ Blue Chip	Peak Irradiance* (mW/cm^2^)
**Bluephase 20i**	Ivoclar	500339	Low, High, Turbo	8 10	7.4 9.0	43.0 63.6	385–515	V/B	650, 1200 & 2000
**Bluephase Style**	Ivoclar	1100001962	Standard	10	9.0	63.6	385–515	V/B	1100
**Bluephase Style M8**	Ivoclar	1130000241	Standard	10	9.0	63.6	430–490	B	800
**TransLux2Wave**	Kulzer	312SF024	Standard	8	7.5	44.2	385–510	V/B	>1400 (Cure Rite)
**Scanwave Prototype**	Acteon	PRT06	tP, PP & CQ	7.5 (Multifiber) 7.5 (Monofiber)	6.9 7.4	37.4 43.0	390–510	V/B	N/A
**S10**	3M	9.3912300E+11	Standard	10	9.6	63.6	430–480	B	1200
**Deep Cure**	3M	9.3312300E+11	Standard	10	9.6	63.6	430–480	B	1470
**Bluephase 16i**	Ivoclar	1674493	Standard	8 13	7.4 11.8	43.0 109.4	430–490	B	1600
**Supercharged Mini LED**	Acteon	302320–025	High	7.5	7.0	38.5	420–480	B	2000
**SK-L036**	Spark	N/A	Standard	8	7.5	44.2	420–480	B	2200
**Cybird XD**	Dentazon	CXD-13142425	High & PE	8	7.2	40.7	430–490	B	1500 & 2700
**S.P.E.C 3**	Coltene	130630030	High & 3K	8 11	7.4 10.0	43.0 78.5	430–490	B	1600 & 3000–3500
**BA Ultimate 1400**	BA International	BA110507	Standard	8	7.0	38.5	400–480	V/B	1400
**Xlite 3**	3H Dental	114000089	Standard	8	6.5	33.2	385–515	V/B	1100
**BLAST lite**	First Medica	BL0446	Standard	8	7.2	40.7	not available	B	2200
**Omega LED**	O'Ryan	L-LS6:0 08080520	Standard	8	7.6	45.4	not available	B	450
**Bluephase C8**	Ivoclar	N/A	Standard	8	7.4	43.0	420–480	B	800
**JAS-2001 B**	Online	N/A	Standard	8	6.6	34.2	400–490	B	>2700
**Demetron A2**	Kerr	95 430	Standard	4 8 11 13	3.7 7.4 9.9 12.5	10.8, 43.0, 77.0, 122.7	450–470	B	1100

*Manufacturers stated.

**Measured light-emitting diameter and calculated area

**Table 2:** Type II LCU optical properties and dimensions.

Light curing unit	Manufacturer	Serial Number	Modes tested	Active diameter** (mm)	Active area** (mm^2^)	Wavelength Range (nm)	Violet/ Blue Chip	Peak Irradiance* (mW/cm^2^)
**Xlite 2**	3H Dental	6700001695	Standard	7.8	47.8	380–515	V/B	1600
**Smartlite Focus**	Dentsply	SLAB01489	Standard	7.5	44.2	430–490	B	1000
**Demi Ultra**	Kerr	786000330	PLS	7.9	49.0	438–485	B	1100–1330
**SiriusMax**	ND	MC-132699	Normal/Xtra	9.1	65.0	430–490	B	1200 & 3000
**Pencure VL-7**	Morita	YD3024	Standard	8.8	60.8	420–480	B	1000
**Pencure VL-10**	Morita	CG5004	M2	8.8	60.8	420–480	B	1000
**Fusion 3.0 Blue**	Dentlight	1AF50505	High	9.6	72.4	420–490	B	2700
**Fusion 3.0 Violet**	Dentlight	9AE42226	High	9.6	72.4	390–430	V	2700
**Fusion 5.0 Blue**	Dentlight	LH80306 SF80306	Standard / Plasma	9.6	72.4	415–490	B	4000 (Plasma)
**Fusion 5.0 Violet**	Dentlight	VH10640 SF80306	Standard / Plasma	9.6	72.4	390–430	V	2700 (Plasma)
**Valo**	Ultradent	V02539	Standard	9.6	72.4	395–480	V/B	1000
**Valo Grand**	Ultradent	S00906	Standard/High/Xtra	11.7	107.5	395–480	V/B	3200 (Xtra)
**Radii Plus**	SDI	31950	Standard	7.0	38.5	440–480	B	1500
**Coltolux**	Coltene	9224025	Standard	7.8	47.8	450–470	B	NA
**FLASH lite Magna**	Den-Mat	9338018	Standard	11.0	95.0	440–490	B	> 1100
**i LED**	Woodpecker	L1731737i	P1 & P2	7.8	47.8	420–480	B	2300 (P1)
**DTE LUX-1**	Woodpecker	L176_7X1	Standard	6.4	32.2	420–480	B	850–1000
**Xlite 4**	3H Dental	C000120B4	Standard	7.0	38.5	385–515	V/B	2000
**Radii Xpert**	SDI	15010	Standard	7.5	44.2	440–480	B	1500

*Manufacturers stated values.

**Measured light-emitting diameter and calculated area. For Type II LCUs the external head diameter is neither relevant nor reported by the manufacturers.

Effective photopolymerization is a significant contributing factor for the quality and long-term clinical success of light-activated resin-based composite restorations. Many factors influence polymerization efficiency, and the LCU is a key extrinsic variable in the final material properties of the photocured resin [4–6]. The complex interaction of light with highly filled photopolymerizable resins [3–5], or even cells and tissues for therapeutic purposes [7], is often not given the sufficient attention that is critically required. For photopolymerizable resins, the fact that the material nearest the light source hardens first begs the question as to what lies beneath. This is an Achilles’ heel of light curing. Ironically, dental practitioners have no accurate and straightforward way of assessing their LCU output and, therefore, of determining appropriate radiation times for their materials [8, 9].

Light meters or dental radiometers (DRs) have been proposed as a better way of monitoring LCU performance compared with simple tactile surface hardness 'tests,' and different conclusions are reported in the literature [10–22]. Many investigators have reported that radiometers provide only limited reliability for accurate irradiance measurements of a range of LCUs [16–22]. For example, Harlow et al. [9] concluded that a single irradiance value derived from a DR or laboratory-grade power meter was inadequate for describing light output and that the spectral output of one broad-spectrum LED-LCU varied over the radiation period. Marović et al. [20] reported that the time point of measurement of two DRs influenced their accuracy relative to an integrating sphere control measurement, whereas Sulaiman [18] found no significant difference for measurement time when testing a different make of radiometer. Price et al. [19] compared the intra- and inter-brand accuracy of four DRs and reported significant differences between the irradiance values recorded for the three examples of each brand tested. In contrast, Leonard et al. [16] found no statistically significant differences in the irradiance values among the five samples of three DRs. Furthermore, Kakeyama et al. [21] reported that irradiance values of twelve different light sources varied with the five tested DRs relative to a laboratory-grade spectroradiometer and attributed the differences to the spectral sensitivity of each radiometer. Shimokawa et al. [22] recently investigated the accuracy of four DRs for measuring the output of nine LED LCUs compared to a fast response laboratory-grade power meter. The authors reported no significant difference (p = 0.527) in accuracy between a radiometer (Bluephase Meter II, BM II) with a purported spectral sensitivity between 380 and 550 nm and the power meter (PowerMax-Pro 150 HD), although the irradiance values reported from the BM II were consistently less (~7%). Ideally, DRs should be capable of estimating power or irradiance values for all makes and models of dental LCUs to within ±20% of absolute values usually measured by a gold standard (GS) integrating sphere. The light detectors of nearly all DRs are based on silicon photodiodes, which have greater responsivity to the longer wavelengths of light, as do photovoltaic cells.

While manufacturers of DRs claim accuracy of their units between ~5–20%, comparisons against GS test instruments (mentioned above) typically reveal differences of ~20–50%. Reported inaccuracies include physical [15, 16], spectral [21], temporal [20] and thermal [20] factors. However, previous investigations have used only small samples of radiometers and LCUs, and meter ranking and accuracy vary widely between studies. A major confounding factor remains the variation in design and radiant output patterns of individual dental LED-LCUs [8, 9]. A simple classification which describes the two principal different designs of LED-LCUs is proposed and presented here: either a Type I unit that uses a light guide (usually a graded optical fibre bundle) for light delivery, or a Type II unit which incorporates the LED chipset directly in the unit head. Light guides of Type I LCUs also vary according to material, taper, and entry and exit diameters. The latter is a critical variable in the radiometer response [16]. Generally, light guides with larger entrance and exit diameters will deliver more power so that their irradiance is acceptable. In contrast, light guides with smaller exit diameters do not need to be so powerful to deliver high irradiance values.

Consequently, the aims of the current work were to:

1. Test the ability of a range of commercial dental radiometers to measure a representative selection of 19 Type I and 19 Type II LED LCUs relative to the “gold standard” laboratory-grade integrating sphere, and fiber-optic coupled spectrometer derived irradiance values.
2. Measure the “snapshot” power output of the LED-LCUs with three examples of a BM II DR and to compare the data with power outputs recorded with a traditional laboratory thermopile sensor instrument (PM10-19C; Coherent) and the fast response power meter (PowerMax-Pro 150 HD; Coherent).
3. Measure the spectral radiant power outputs of the Type I and Type II LCUs over the entire exposure period with a benchtop fiber-coupled spectrometer instrument (MARC-LC, BlueLight Analytics) and to compare this method with the “GS” radiometric test.
4. Determine whether the dental DRs are less accurate in recording irradiance for broad-spectrum LED-LCUs, given that the spectral response of silicon photodiode sensors varies between violet, violet-blue and blue wavelength bands.

The null hypotheses of this study were:

1. There will be no significant difference (P>0.05) between the mean pooled radiant power values measured with the integrating sphere and fiber-coupled spectrometer “GS” test method and a radiometer with wide spectral sensitivity (~380–550 nm; BM II, Ivoclar-Vivadent), for either Type I or Type II LED-LCUs.
2. There will be no significant difference (P>0.05) between radiant power values measured with a laboratory-grade thermopile sensor (PM10-19C, Coherent) and a fast response thin-film detector (PowerMax-Pro 150 HD).
3. There will be no significant difference (P>0.05) between radiant power values measured with the “GS” test method and the mean of the power values recorded over the entire exposure duration as recorded with a benchtop calibrated instrument (MARC-LC, BlueLight Analytics).
4. No tested commercial DR will be within ±20% of the mean irradiance value derived using the “GS” test method.
5. All tested commercial DRs will give significantly lower (P<0.05) normalized irradiance values for LCUs where the majority (>60%) of spectral power is <450 nm in comparison with those lights where most (>65%) of the spectral radiant output is >450 nm.

## 2. Materials and methods

The commercial DR models under investigation are described in Tables 1 and 2 according to the manufacturer, model, serial number, display type, sensor window aperture diameter, irradiance recording, and spectral sensitivity ranges. The Bluephase Meter I (BM I) and the Ledex CM 4000 are the only radiometers which use linear sensors.

The light output of nineteen Type I (Table 1) and nineteen Type II LCUs (Table 2) was evaluated. A number of the units were tested in different power and/or spectral radiant power combinations. Five Type I units were also tested with light guides of different exit diameters or configuration, resulting in 61 test groups. The Type I ‘Scanwave’ (Satelec, Acteon, France) was a prototype unit that, unlike the commercially marketed variant (Mini-LED Scanwave), allowed continuous light output in three different spectral ranges depending on which pair of the four different wavelength chips were activated. This unit was tested both with a standard multifilament tip as well as a solid (single filament) glass tip, which distributed the light output more uniformly across the tip exit face. Six of the Type I and Type II LCUs incorporated some or all of their light output in the violet range (Tables 1 and 2).

### 2.1 Spectrometer measurements

Two spectrometer systems were compared, namely:

#### 2.1.1 A “GS” integrating sphere system

A 15 cm diameter laboratory-grade integrating sphere (Labsphere, North Sutton, NH USA) with a custom 16 mm entrance port linked to a calibrated fiber-optic coupled spectrometer (USB 2000+, Ocean Optics) was used to record total power in a “snapshot” fashion, ~1 s after the light was activated, within the 380–540 nm range, and spectra for each LED-LCU (n = 3). An internal reference light source (Labsphere) was used to calibrate the system before the measurements. The percentage of the power output for each LCU in the 380–450 nm range and the 450–520 nm range was also determined. The current international standard for testing dental powered polymerization activators (ISO 10650–2018) has added a method for measuring radiant exitance (section 7.4.1) using a spectrometer and compatible Integrating Sphere assembly to the previous method (section 7.4.2) which used bandpass filters in conjunction with a flat response power meter.

#### 2.1.2 A benchtop calibrated instrument

The MARC LC^TM^ system (Bluelight Analytics, Inc., Halifax, Canada) featured an upper sensor with a light collection chamber, incorporating a sealed diffuser window overlying the 16 mm entrance port of a two-inch integrating sphere linked to a USB 4000 spectrometer (Ocean Optics). The system is calibrated by the manufacturer using a HL 2000 calibration lamp (Ocean Optics). Spectral radiant power measurements were recorded continuously over the entire radiation time using the manufacturer’s custom software. This allowed mean and maximum power values to be recorded in addition to radiant power spectra.

### 2.2 Laboratory-grade power meter measurements

Two laboratory-grade power meters, a traditional thermopile sensor (PM10-19C; Coherent, Ely, UK), and a fast response thin-film detector (PowerMax-Pro 150 HD; Coherent) were used to record power outputs for the LCUs (n = 3) at 10 Hz. The emitted radiant power of each LCU was measured with the LCU light source exit tip or window fixed ~0.5 mm from, but without touching, the detector surface. Power output was recorded continuously, and the peak power output was registered. For the PowerMax-Pro, LCU power was also measured with the 20 kHz (high-speed) acquisition rate to resolve any temporal pulse output pattern for each LCU and to eliminate the effect of aliasing on average power readings with LCUs that exhibited a high pulse repetition rate.

The internal diameters corresponding to the “effective” or optically active light-emitting exit window diameters of the Type I and Type II LCUs were measured to the nearest 0.1 mm with a digital micrometer (Mitutoyo Inc., Japan) and confirmed with a digital microscope (Veho, Dayton, OH, USA). Irradiance values for each LCU were then derived from the radiant power measurements using the quotient of the radiant power and the calculated light source emission area.

### 2.3 Bluephase Meter II power measurements

Three examples of the BM II (Table 3) were used to record power measurements for each LCU (n = 3 per LCU). Light guide or light exit window surfaces were positioned as directed by the manufacturer’s instructions, i.e., in contact with the meter surface.

**Table 3:** The radiometer models tested are listed according to the manufacturer, model, sensor aperture diameter, irradiance, and spectral sensitivity range.

Manufacturer	Model	Serial Number	Analog / Digital	Sensor Window Aperture (mm)	Irradiance Range (mW/cm^2^)	Wavelength (nm)
**Demetron**	L.E.D.	79309372	Analog	7	< 2000	400–500
**S.D.I**	LED	2–22481	Digital	12	< 2000	400–525
**Ivoclar**	Bluephase Meter I (BMI)	5667	Digital	3	300–2500	385–515
**Ivoclar**	Bluephase Meter II (BMII)	1300000003; 1300001134; 1300001150	Digital	15	300–12000	380–550
**Tpcdental**	TPC LM-300 LED & QTH	L143000296	Digital	7.25	0–2500	400–500
**Efos**	Cure Rite 8000	3594	Digital	10	0–2000	400–525
**Dentsply**	Cure Rite 644726	7886	Digital	6.5	0–2000	400–525
**Coltene**	Coltolux C7900	95101064	Digital	8.5	0–2000	400–525
**Mega-Physik**	Cromatest 7041	70410366	Digital	9	100–1999	400–525
**Dentmate**	Ledex CM 4000	CB0520	Digital	2	200–4000	390–500
**Rolence**	Meter 200	1317/2009	Digital	6	0–5000	400–520
**CMS**	CQ LIT	2013	Analog	6	0–6000	N/A
**3H**	LED	N/A	Digital	8.5	0–2000	350–550
**Holz**	LED	C00000CC2	Digital	8	50–3600	350–550
**Woodpecker**	LM-1	541004	Digital	8	0–3500	400–500
**FVE**	BTM-2000	FVB001415	Digital	10	0–999	400–540

Irradiance and wavelength ranges are drawn from manufacturers’ literature. The two underlined meters BM I and Ledex CM 4000 are the only ones that use linear sensors. Of the tested models, the BM II (n = 3) (Ivoclar-Vivadent, Liechtenstein) is the only radiometer that can measure power. It can also calculate the irradiance if the light probe has a 6 to 12 mm circular diameter.

### 2.4 Dental radiometer irradiance measurements

Three readings were taken for each of the LCUs in each test condition, i.e., 'modes tested' and 'tip type and diameter’ (Tables 1 and 2) using the hand-held DRs listed in Table 3. Two of the DRs (Ledex CM 4000 and BM I) incorporate linear strip sensors, 2 mm and 3 mm in width, respectively, that are designed to estimate light output tip diameter to calculate LCU irradiance. For the BM II, the scale on the underside of the instrument allowed the round exit tip external diameters from 6 to 12 mm of the Type I LCUs to be estimated to the nearest 1 mm for irradiance measurement. GS irradiance of the Type II LCUs was calculated from power measurements using the ‘active light-emitting areas’ (Table 2). All the irradiance values for each LCU and radiometer combination were normalized relative to the mean irradiance determined for the LCU from the “GS” test method (Section 2.1.1), which allowed differences to be determined for each light source, mode and tip combination. Fitted line regression plots with 95% confidence and prediction intervals were generated to assess the power meter and DR results using the GS as the predictor variable.

### 2.5 Dental radiometer spectral response measurements

A light source (AURA, Lumencor, USA; optically active (light-emitting) diameter = 5 mm) with three spectral band outputs (λ_max_ = 405 nm, 470 nm, and 550 nm) was calibrated to deliver 957 + 23 mW/cm^2^ in the blue, 973 + 10 mW/cm^2^ in the cyan and 1218 +1 mW/cm^2^ green spectral bandwidth (pooled mean 1049 ± 146 mW/cm^2^) and was used to assess the spectral response of each radiometer under investigation. For each band, a calibration curve was produced using a USB4000 spectrometer (Ocean Optics) coupled with a 200 μm fiber and 3.9 mm diameter cosine corrector (CC3; Ocean Optics) in OceanView (Ocean Optics). Prior to measurements, the spectrometer set-up was calibrated using a broadband deuterium-tungsten calibration lamp (DH2000, Ocean Optics). The tip of the liquid light guide was aligned centrally with the cosine corrector and measurements were taken at 0mm separation for light source power settings of 2–100%. Following regression analysis, power settings to deliver ~1000 mW/cm^2^ were determined for each spectral band (22%, 48% and 26% for 405 nm, 470 nm, and 550 nm, respectively). The calibration data is reported in S2 Fig. This test was used to complement the findings from Section 2.1.1 regarding the spectral sensitivity of the DRs in the wavebands 360–450 nm and 450–540 nm.

### 2.6 Beam profile measurements

Beam profile images were recorded for the LCUs using a CCD array laser beam profiler camera placed at a fixed distance from the diffusing surface of a translucent frosted glass screen target (DG2X2-1500, Thor Laboratories, Newark, NJ, USA). The light tip or exit window was placed against the target diffusing surface to record the images. Details of the method have been described previously [8]. Briefly, each image was individually calibrated, according to the power recorded for each LCU with the integrating sphere by setting the highest level measured to assume the highest array count in the BeamGage Professional (v5.6: Ophir Spiricon) software. For this work, a custom filter (International Light Technologies, Peabody, MA, USA) was also used to flatten the camera's spectral response. The Ultracal^TM^ feature of the BeamGage software was used to correct for ambient light. The mean emitted radiant power values obtained by the IS method were input to the BeamGage software to produce color-coded calibrated 2D images of irradiance distribution for the LCUs. Additionally, beam profile images of two single spectral peak or so called “monowave” (S10 and Cybird) and one multi-peak or so called “polywave” LED-LCU (Bluephase Style) were also made (a) with the camera lens focused directly onto the light guide tip exit face (b) through the mirrored attenuator of a disassembled BM II radiometer and (c) through the assembled optics (housing aperture, diffusers, and filters) of a disassembled Demetron L.E.D. radiometer. The BM II front surface mirror attenuator and the photodetector both were greater in size than any tested LCU emission area. In contrast, the Demetron L.E.D. radiometer had a typical small area photodiode detector (see Fig 2B).

### 2.7 Statistical analysis

Linear regression analysis (Minitab v18 software) was used to investigate the relationship between the power data determined by the BM II radiometer, the laboratory-grade power meters, and the GS integrating sphere assembly test method. Similar analyses were performed for irradiance data derived from the power measurements using the GS test method and the tested commercial DRs. Parametric analysis of variance methods (balanced and general linear model methods) were used to compare raw data at P = 0.05 significance level. Distribution free Mann-Whitney U tests were employed to compare the spectral responsivity of the commercial radiometer models to the GS test method using the normalized irradiance data generated by two subsets of LCUs where the majority of the spectral output was either above (>65%) or below (>60%) 450 nm.

## 3. Results

### 3.1 Power and irradiance data

**Fig 1:**
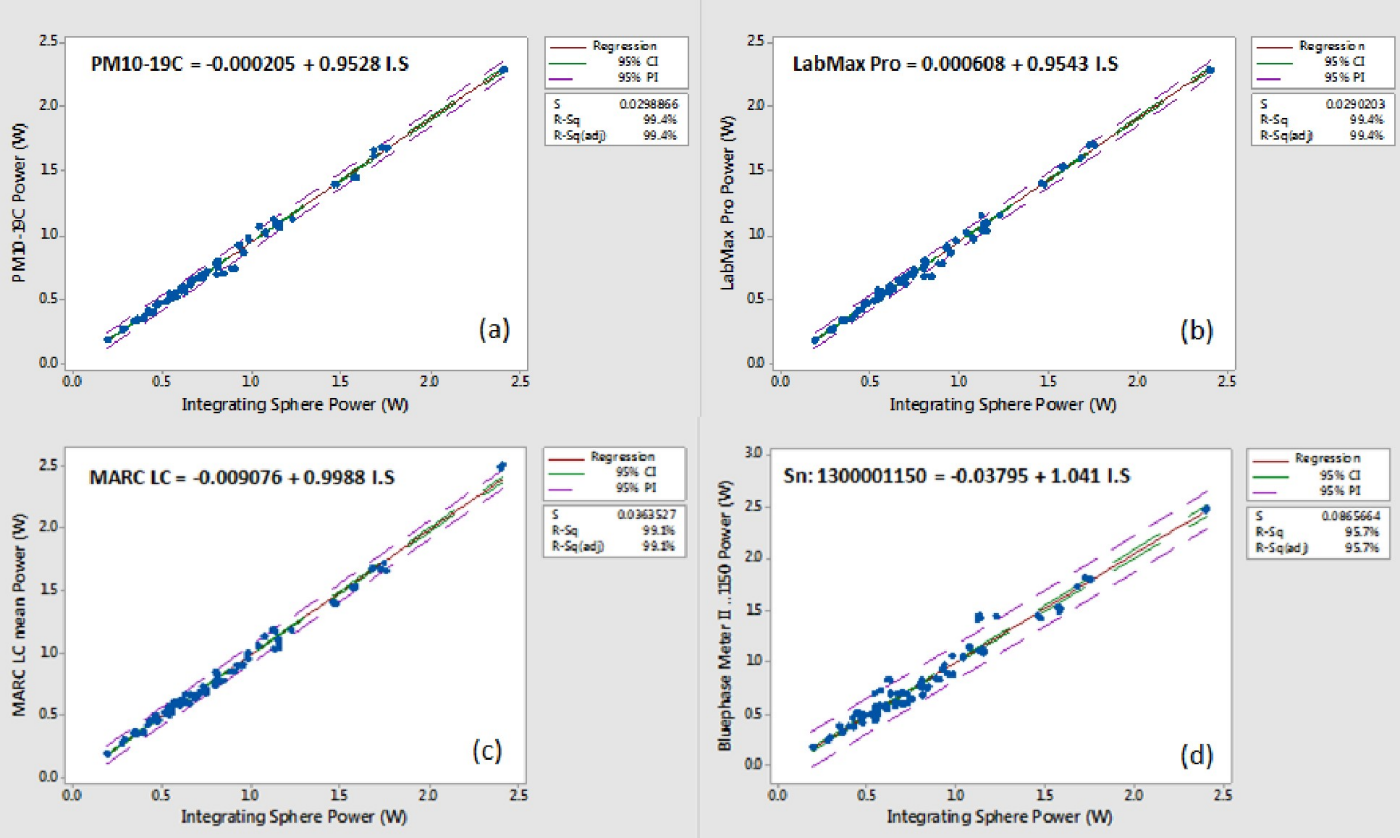
Regression analyses and fitted line plots with 95% confidence and prediction intervals for mean power recordings of the Type I and Type II LED-LCUs using (a) the PM10-19C thermopile, (b) the LabMax Pro, (c) the MARC LC and, (d) the BMII (sn: 1300001150). For each analysis, the integrating sphere gold standard power (W) data was used as the predictor variable. Several high outlier results were noted in Fig 1 (d) for Type II LED-LCUs (I LED in standard and high output modes, Pencure VII and Fusion 5 (Blue Head) in Plasma mode). As the BM II is only designed to generate irradiance data for light guide tip diameters between 6 and 12 mm, the values generated for the 4 mm and 13 mm tips of the Demetron A2 LED LCU were based on programming the nearest tip diameter value (6 mm and 12 mm, respectively) to obtain the readings which were then corrected for area differences.

Individual power values recorded using GS integrating Sphere system ranged from a low of 0.19W for the Omega LED-LCU to a high of 2.40W for the Xtra Power mode of the Valo Grand. Excellent agreement (R^2^ adj. values > 99%) was found when the two laboratory-grade power meters (PM10-19C and PowerMax-Pro 150 HD) and the benchtop calibrated instrument (MARC LC) were compared with the integrating sphere mean power values as the predictor variable, thus confirming the accuracy of the GS test data (Fig 1A–1C). When additional regression analyses were performed based on Type I and Type II LED-LCUs separately R^2^ adj. values remained above 99% for all three control power measurement instruments. Regression R^2^ adj. values for power readings recorded with the three examples of the BM II radiometer compared with the integrating sphere GS power data were lower with wider confidence and prediction intervals but still above 95% for the combined Type I and II LED-LCU power data (Fig 1D).

**Fig 2:**
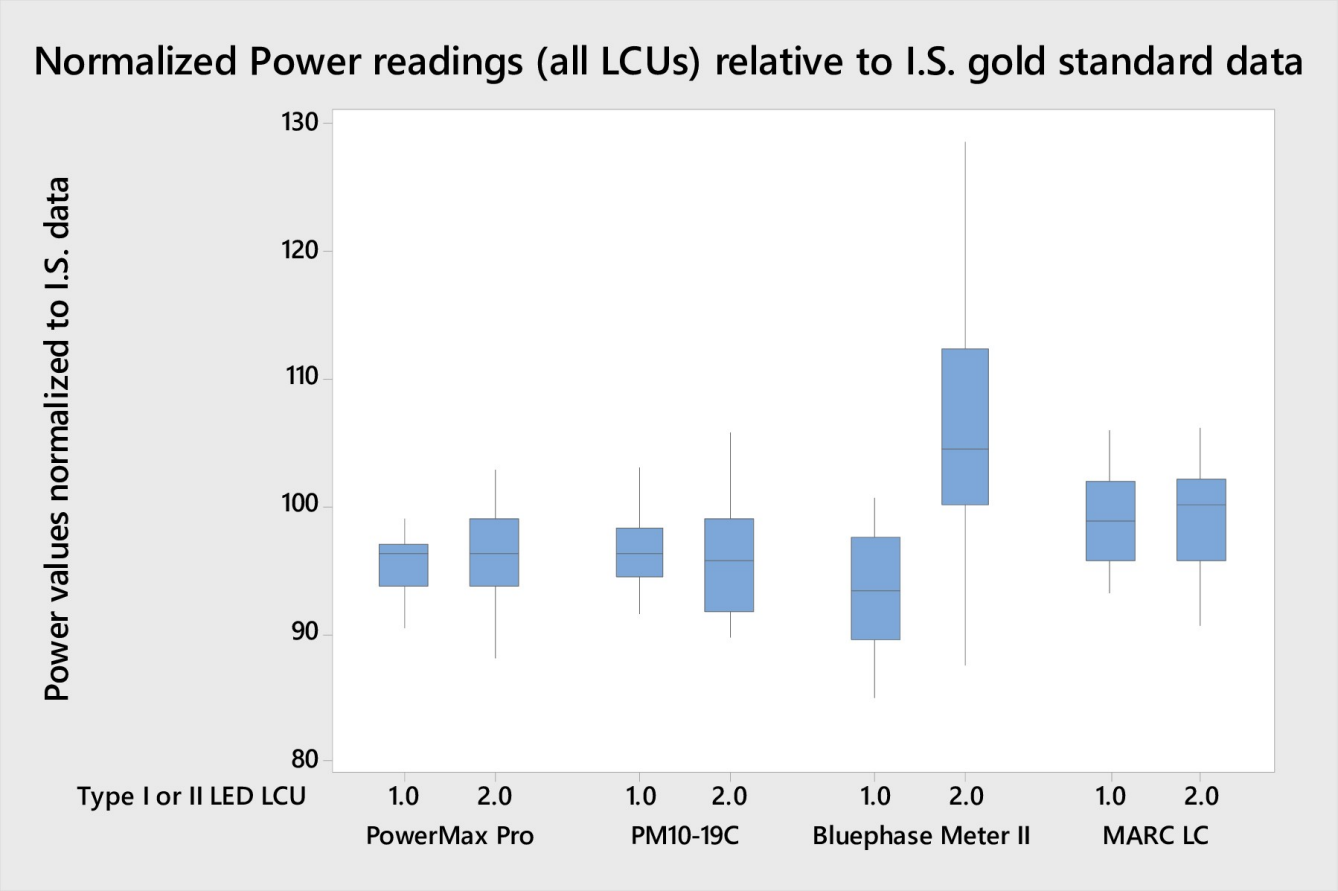
Pooled normalized mean power readings for all LED-LCUs relative to the integrating sphere gold standard data 100% values. Note the difference between the Type I and Type II LED-LCU data distributions for the BM II units (average of the three tested units shown) compared with the two laboratory-grade power meter instruments and the MARC LC integrating sphere / spectrometer apparatus.

No statistically significant difference (P>0.05) in the power values from the LCUs were recorded for the three examples of the BM II. The mean pooled difference between individual LCUs compared by any two of these three meters was < 2% showing excellent intra-meter power reading reproducibility. For the three BM II instruments, overall power values for Type I LED-LCUs were 6.9% lower than the GS. However, the mean pooled normalized results for Type II LCUs tested with the BM II instruments ranged from 6.7 to 8.3% greater than the GS values (Fig 2), and the difference was statistically significant (P < 0.001; DF = 24). Power data for Type II LCUs recorded with the PM10-19C and PowerMax-Pro were 4.1% and 3.8% lower than the GS value, respectively; a pattern similar to the results for the Type I LCUs. Individual Type II LCUs (Smartlite Focus, Pencure VII, Fusion 5.0, and i LED) generated power readings >10% above the corresponding GS test method data for all three tested examples of the BM II. For three LCUs (Pencure VII, Fusion 5.0, and i LED) the mean readings were >25% above the GS. Power readings taken with the MARC LC yielded results closest to the GS test method with normalized pooled values that were 1.2% and 0.5% lower for Type I and Type II LCUs, respectively.

**Fig 3:**
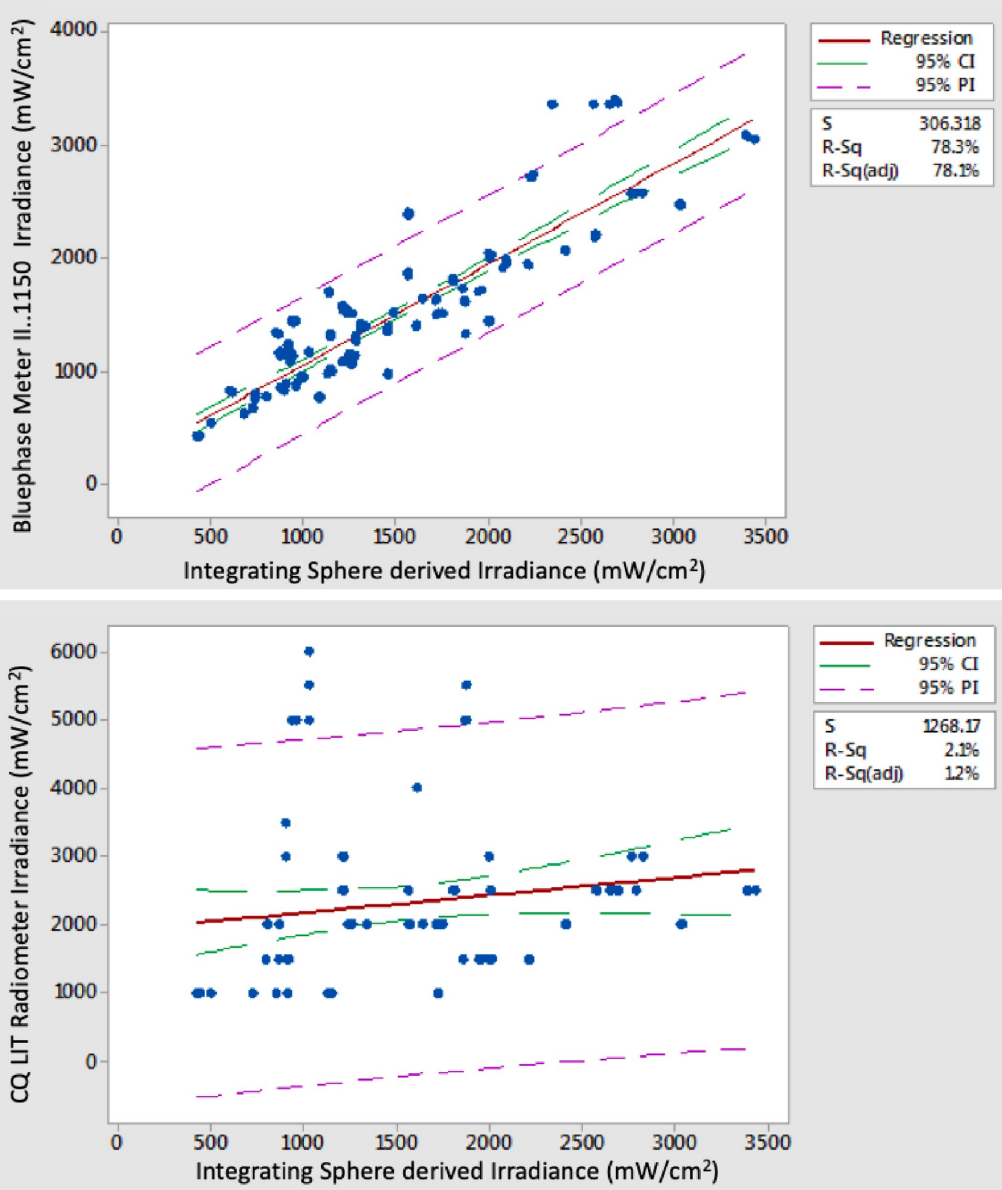
Regression analysis fitted line plots comparing irradiance data for the (a) BMII (sn:1300001150) and (b) the CQ LIT radiometer with the corresponding Integrating Sphere derived irradiance data sets as the independent variable.

**Fig 4:**
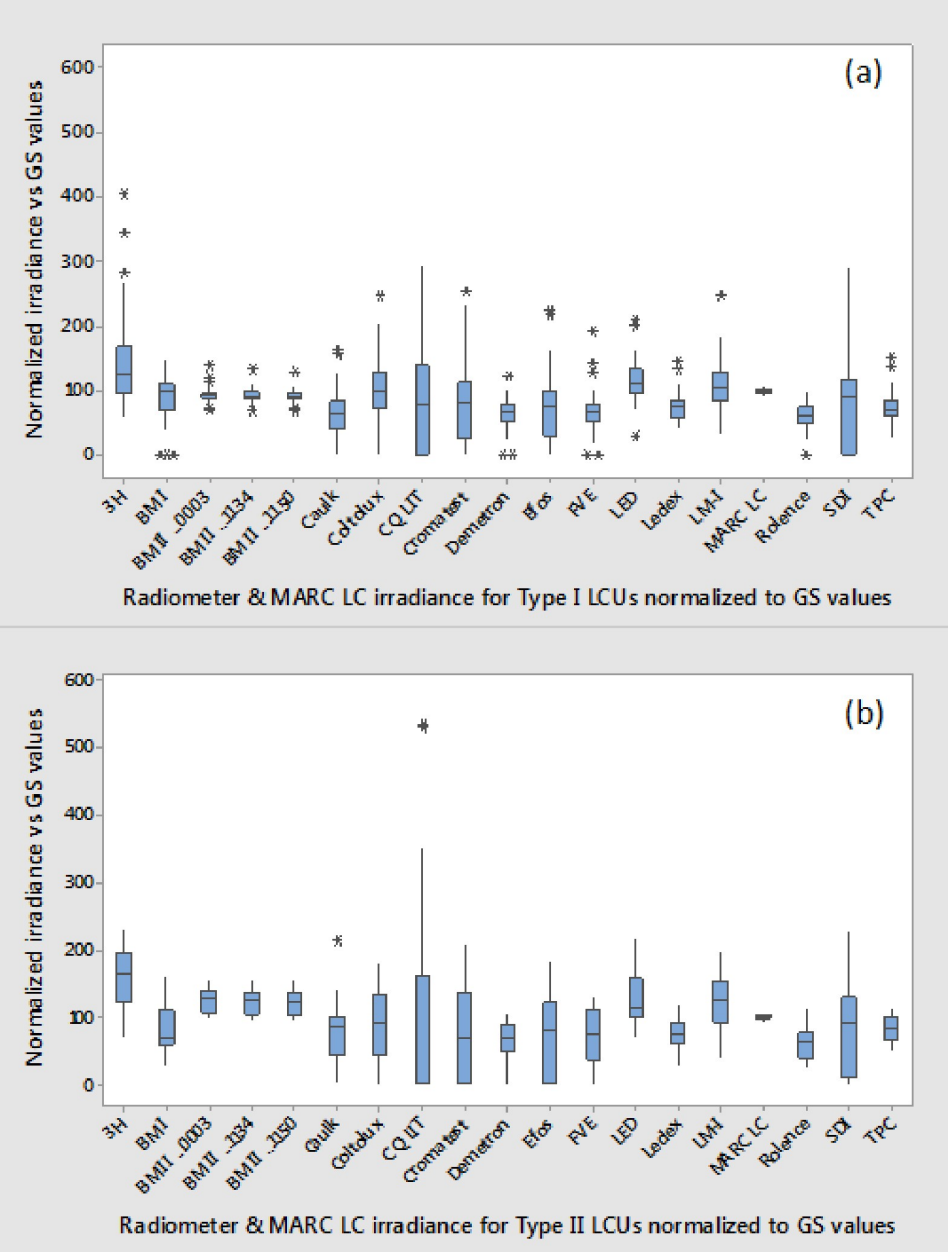
Normalized pooled mean irradiance for all LCUs; (a) Type I and (b) Type II tested with the sixteen models of dental radiometers—the 3 examples of the BM II are denoted by the last 4 digits of their respective serial numbers …0003, …1134, …1150 and the MARC LC instrument (calculated from mean power data) relative to the gold standard Integrating Sphere method. Note the extremely wide data spread for many radiometers.

Agreement (R^2^ adj. values) between irradiance data for the sixteen models of radiometer versus the IS “GS” derived mean irradiance values for the combined data from Type I and Type II LED LCUs was significantly lower than power comparisons ranging from a high of 77.1–78.1% for the BM II devices to a low of 1.2% for the CQ LIT radiometer (Fig 3). High outliers above the prediction interval line caused by several LCUs were noted in the fitted line plots of the power (Fig 1D) and irradiance data for the three tested examples of the BM II radiometers (Fig 3A). They were related only to Type II LED LCUs. This reflects the value of testing a wide range of radiometers and LCU combinations as would be encountered in clinical practice. In contrast, irradiance readings derived from mean power recordings taken with the MARC LC were within an average of 0.8% of the corresponding integrating sphere GS test method data (Figs 1C and 4).

**Fig 5:**
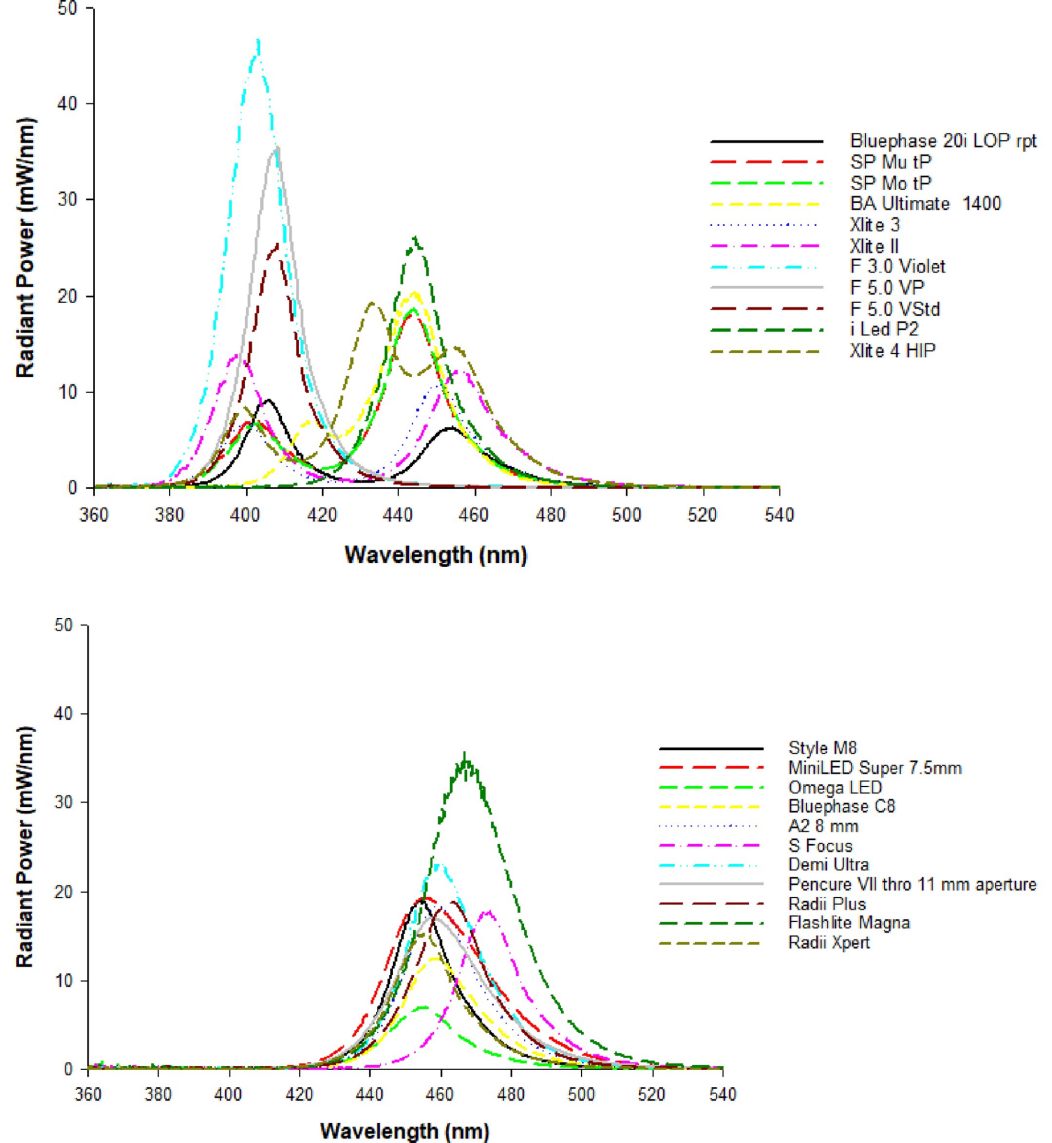
Radiant power of the LCUs, that delivered a) greater than 60% of their power output below 450 nm and b) greater than 65% of output above 450 nm.

Based on the results, the:

1. The first null hypothesis was accepted as there was no significant difference between the pooled mean power value recorded for the GS ((Type I LCUs pooled mean 0.6988 W (95% C.I. 0.6422–0.7554) and Type II LCUs pooled mean 0.8251 W (95% C.I. 0.3404–2.4068)) and the BM II (Type I LCUs pooled mean 0.6487 W (95% C.I. 0.5921–0.705) and Type II LCUs pooled mean 0.8896 W (95% C.I. 0.3743–2.508)) for either type I (P = 0.219) or type II (P = 0.423) LCUs. When individual LCU mean power values were compared however, significantly greater mean power values (P<0.05) were recorded for GS versus BM II for all but one type I LCU (JAS-2001 B) which showed no significant difference (P>0.05) between GS and BM II. In contrast, when individual LCU comparisons were made for type II LCUs fifteen units registered significantly greater mean power (P < 0.05) when tested by GS in comparison to BM II and only four LCUs (Demi Ultra, Valo, Coltolux and Radii Expert) the opposite. As a consequence, the pooled mean power values for the BM II normalized to the GS test method were significantly higher for Type II versus Type I LCUs (P<0.05; Fig 2). Further, there was no significant difference (P>0.05) between the response of Type I and Type II LCUs for either of the two laboratory-grade power meters or the MARC LC instrument (Fig 2).
2. The second null hypothesis was accepted as there was no significant difference (P = 0.856) between mean power readings recorded with the PM10-19C thermopile instrument and the fast response PowerMax-Pro sensor (Fig 2).
3. The mean power for all LCU tests recorded using the MARC LC was 0.743 W (95% CI: 0.686–0.800), and for the GS test method, it was 0.753 W (95% CI: 0.696–0.810). There was no significant difference between the means (P = 0.807), and therefore, the null the third null hypothesis was accepted.
4. All three examples of the BM II produced an average irradiance deviation from the GS test method of between 16.4–17.4%. Therefore, the fourth null hypothesis was rejected in part. However, the remaining radiometers produced greater mean deviations from the GS, ranging from 23.2% (BM I) to 84.5% (CQ LIT radiometer; Fig 3). Fig 4 highlights the substantial deviation in irradiance data for the sixteen models of radiometer relative to the IS control and the MARC LC instrument. Ten of the radiometers failed to give any reading with one or more LCU. The CQ LIT instrument was unable to provide any reading in one or more test conditions, with 20 of the 38 tested LCUs. Individual LCU and radiometer combinations produced mean irradiance values ranging from 7% (Fusion 5.0, “Plasma” output mode tested with the SDI radiometer) to 535% (Smartlite Focus tested with the CQ LIT radiometer) of the GS normalized value.
5. Fig 5A and 5B highlight the spectra for those LCUs and operation modes having >60% of their output below or >65% above 450 nm, respectively (S1 Fig provides the radiant power exitance for all the tested LED LCUs). Eight LCUs in one or more modes had >60% of their output below 450 nm, and eleven had >65% of their output above 450 nm. Normalized data for these two subsets of lights were compared with Mann Whitney U-tests. LCUs with a majority of violet output had significantly lower (P<0.05) normalized median responses (relative to the GS) compared with the LCUs with a majority of blue light output for most radiometers. Consequently, the fifth null hypothesis was accepted in part. However, the BM I and the three tested examples of the BM II exhibited no significant difference (P>0.05) between medians; thus, these four radiometers behaved differently from the other dental radiometers.

### 3.2 Radiometer spectral response measurements

A further series of tests were performed with a calibrated light source (AURA, Lumencor) with three waveband outputs (λ_max_ 405 nm, 470 nm, and 550 nm) to assess the spectral responses of the DRs (Table 4). Seven of the radiometers (BM I & II, TPC LM-300, Ledex CM 4000, CQ LIT, 3H LED and Woodpecker LM-1) had their peak response to green waveband light (λ_max_ 550 nm; Table 4) while the remainder had their peak response to blue output light (λ_max_ 470 nm).

**Table 4:** Mean irradiance readings (n = 3) for the radiometers generated using the Lumencor Light Engine (LE) with irradiance set at 1049 ± 146 mW/cm^2^ for the separate violet (λ_max_ = 405nm), blue (λ_max_ = 470nm) and green light (λ_max_ = 550nm) outputs three wavebands.

Light Meter	LE 405 nm	LE 470 nm	LE 550 nm
**Kerr LED**	100.0 (0)	376.7 (5.8)	0.0 (0)
**S.D.I. LED**	0.0 (0)	413.3 (5.8)	0.0 (0)
**Bluephase Meter I**	830.0 (0)	723.3 (5.8)	850.0 (0)
**Bluephase Meter II**	521.1 (16.2)	482.2 (4.4)	896.7 (10)
**TPC LM-300**	420.0 (0)	510.0 (0)	670.0 (0)
**Efos Cure Rite**	115.7 (1.2)	431.3 (2.5)	60.7 (4.7)
**Caulk Cure Rite**	39.7 (0.6)	389.7 (0.6)	19.7 (0.6)
**Coltolux**	226.7 (0.6)	520.0 (0)	250.0 (0)
**Cromatest**	83.0 (0)	463.3 (0.6)	202.0 (0)
**Ledex CM 4000**	460.0 (0)	776.7 (2.9)	2633.3 (7.6)
**Rolence meter 200**	79.3 (1.5)	406.0 (2.6)	0.0 (0)
**CQ LIT**	0.0 (0)	0.0 (0)	6000 (0)
**3H LED**	317.7 (2.3)	1291.7 (2.3)	1868.7 (2.3)
**FVE BTM-2000**	0.0 (0)	274.7 (0.6)	0.0 (0)
**Woodpecker LM-1**	175.0 (0)	550.0 (0)	1225.0 (0)
**Holz LED**	0.0 (0)	317.3 (1.5)	0.0 (0)

The BM II readings are based on the average of the three tested examples of this radiometer. The discrepancy between the programmed irradiance for the LE and the values here is attributable to the fact that the light engine liquid-filled light guide's external diameter is 2 mm greater than the active light-emitting diameter. When the irradiance is calculated based on the smaller output area the BM II values for the violet and blue wavebands approximated the programmed 1,000 mW/cm^2^ irradiance

A supplementary investigation was conducted on a retrieved mirror attenuator from one of the BM II instruments (sn: 1300001134). Light transmission was measured through the attenuator and without it using the λ_max_ = 405 nm and 470 nm wavelengths of the Aura Light Engine, and ~40% higher transmission of violet was recorded compared with the blue wavelength range. Distribution free Mann-Whitney U tests were used to compare the spectral responsivity of the commercial radiometer models with the GS test method using the normalized irradiance data generated by two subsets of LCUs (Fig 5) where the majority of the spectral output was either below (>60%) or above (>65%) 450 nm. For both BM I and BM II, there was no significant difference (P>0.05) in the normalized pooled median power outputs for the two subsets of LCUs. This was in contrast to all the other 14 DR models tested where the normalized power values were significantly lower (P<0.05) for the violet compared with the blue output range. The flatter spectral response of the Bluephase meters compared with the other radiometers was noteworthy. This indicates a more accurate spectral response for LCUs with multiple LEDs that emit both violet and blue wavelengths [23, 24].

### 3.3 Interaction of light curing units and radiometer optics

The minimum and maximum power values recorded with the GS Integrating Sphere test setup were 0.19 W for the Omega LED-LCU and 2.40 W for the Valo Grand in Plasma output mode. The BM II mean power values for the SmartLite Focus Type II LCU were 12 to 13% higher than the mean integrating sphere control value and 20 to 21% higher than the PowerMax-Pro value.

**Fig 6:**
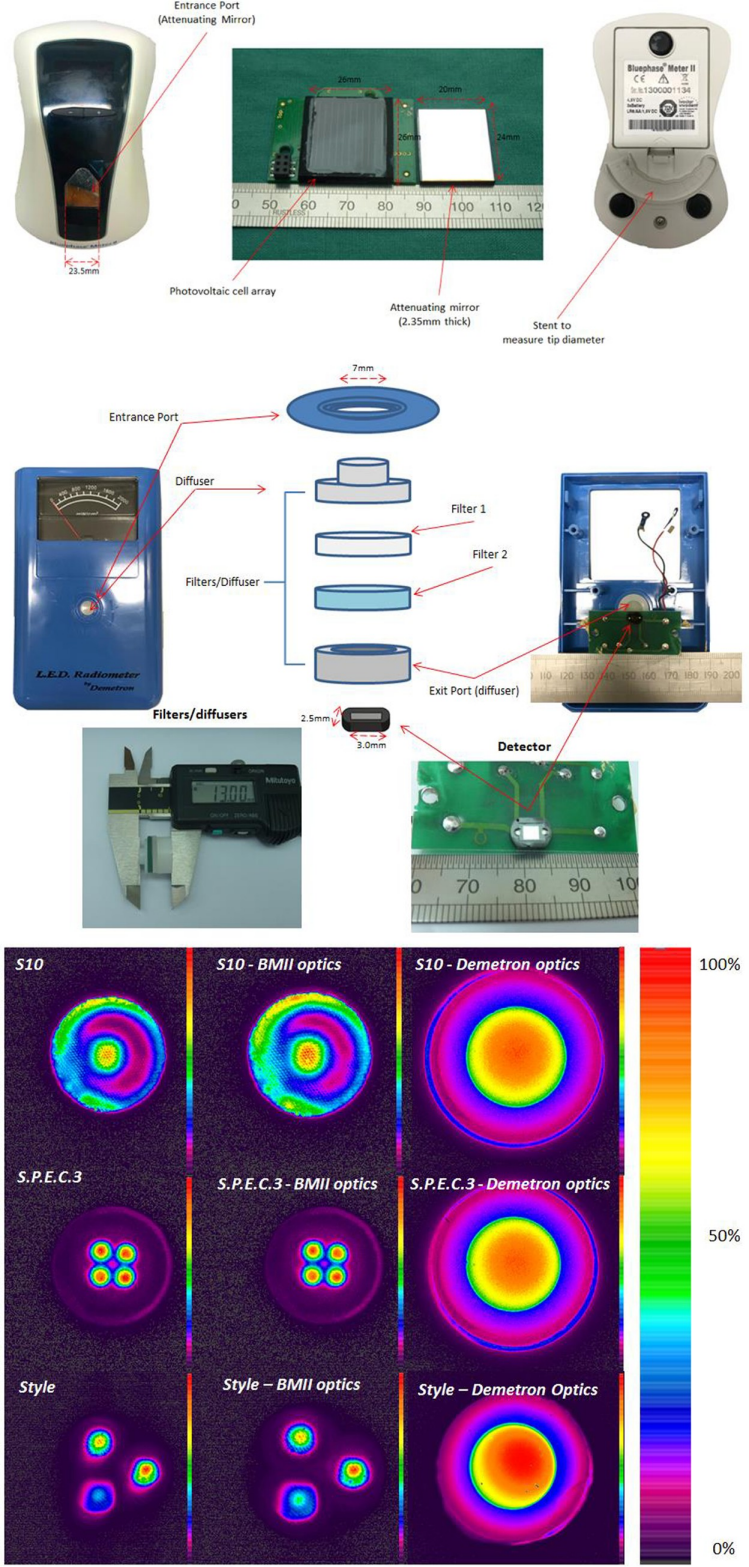
(a) The front surface mirror attenuator from the disassembled BM II (sn:1300001134) and the large area photovoltaic cell array light sensor which lay directly beneath it; and (b) the "optics" components (diffusers, heat and bandpass filters) from a disassembled Demetron L.E.D. radiometer and the small area photodiode sensor typical of other dental radiometers for comparison. The BM II meter incorporates a light tip diameter guide, allowing the operator to measure the light guide tip diameter for programming the instrument in "irradiance reporting" mode. (c) Beam profile images of 2 single spectral peak Type I LED LCUs (S10 and S.P.E.C.3) and one LED LCU with 2 wavelength band outputs (Bluephase Style with the original non-homogenizing light guide to show the two blue and one violet LED chips seperately). From left to right the profiles were recorded by imaging: 1, directly onto the light guide exit face (left column); 2, through the mirror attenuator of the BM II (middle column) which was disassembled after the remainder of the project was completed (Fig 6A; sn: 1300001134); and, 3, through the reassembled optics (Fig 6B) of the Demetron L.E.D. radiometer (right column). Note the relative increase in output for the violet chip (~7 o’ clock position on the image) versus the two blue chips of the Bluephase Style when imaged through the BM II mirror attenuator (middle column–bottom image) compared to imaged directly onto the light guide exit face (left column).

The optics of the disassembled BM II mirror attenuator (Fig 6A) and Demetron L.E.D. radiometer (Fig 6B) were used to image the beam profiles of selected LCUs (Fig 6C). Beam profiles demonstrated that the light was diffused by the Demetron L.E.D. radiometer optics. In contrast, the light pattern was essentially unaltered as imaged through the BM II mirror attenuator for the two monowave LCUs. The output from the violet chip of the Bluephase Style "polywave" LCU was increased relative to the corresponding blue light output when imaged through the BM II mirror, in contrast, to directly imaging the light tip exit face (Fig 6C). In this study, all power and irradiance data for the Bluephase Style was recorded using the unit with the current beam homogenizing tip. The original non-homogenizing tip was only used for the beam profile work in Fig 6 to show the effect of the BM II mirror on the violet and blue chips output separately.

**Fig 7:**
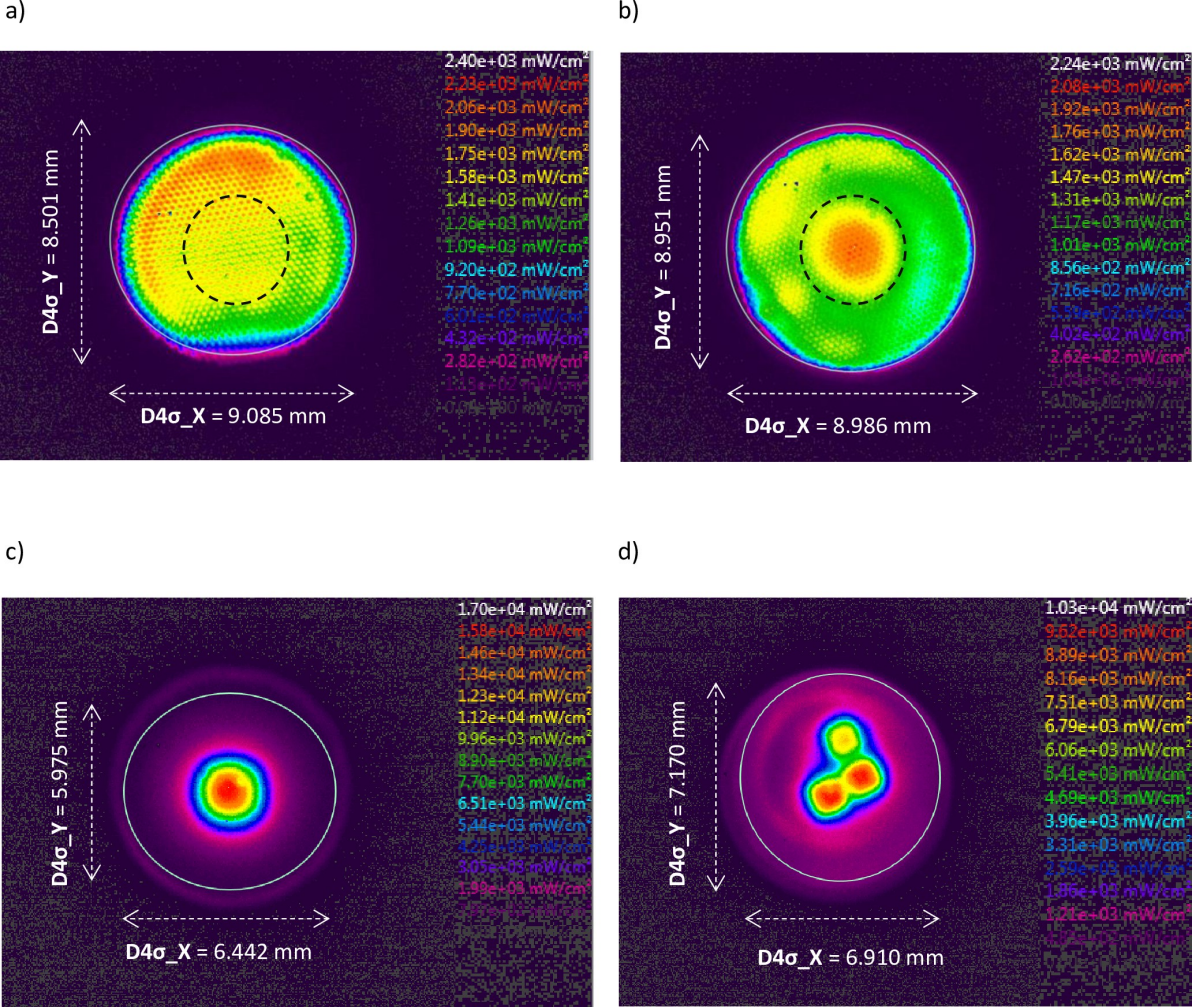
Beam profiles of the (a) Elipar Deep Cure and (b) Elipar S10 units. The beam widths in both the horizontal and vertical directions (x and y, respectively) are given by D4σ which is 4 times σ, where σ is the standard deviation of the horizontal or vertical marginal distribution respectively. Note the more uniform color-coded irradiance distribution across the exit window face of the Deep Cure light guide compared to the central "high spot" peak irradiance region for the S10. The dotted lines represent the beam area that faced the 4 mm cosine corrector of the MARC LC lower sensor. The images explain why the irradiance ranking for these two LCUs reverses when the irradiance is measured over the central 4 mm diameter region of the beam in comparison with irradiance calculated from the total power output related to the optically active area of the light guide (9 mm diameter). Fig (c) and (d) show the local irradiance distributions for the Satelec Supercharged and Spark SK-L036A units, respectively. Readers should note that these images use different irradiance scales.

**Fig 8:**
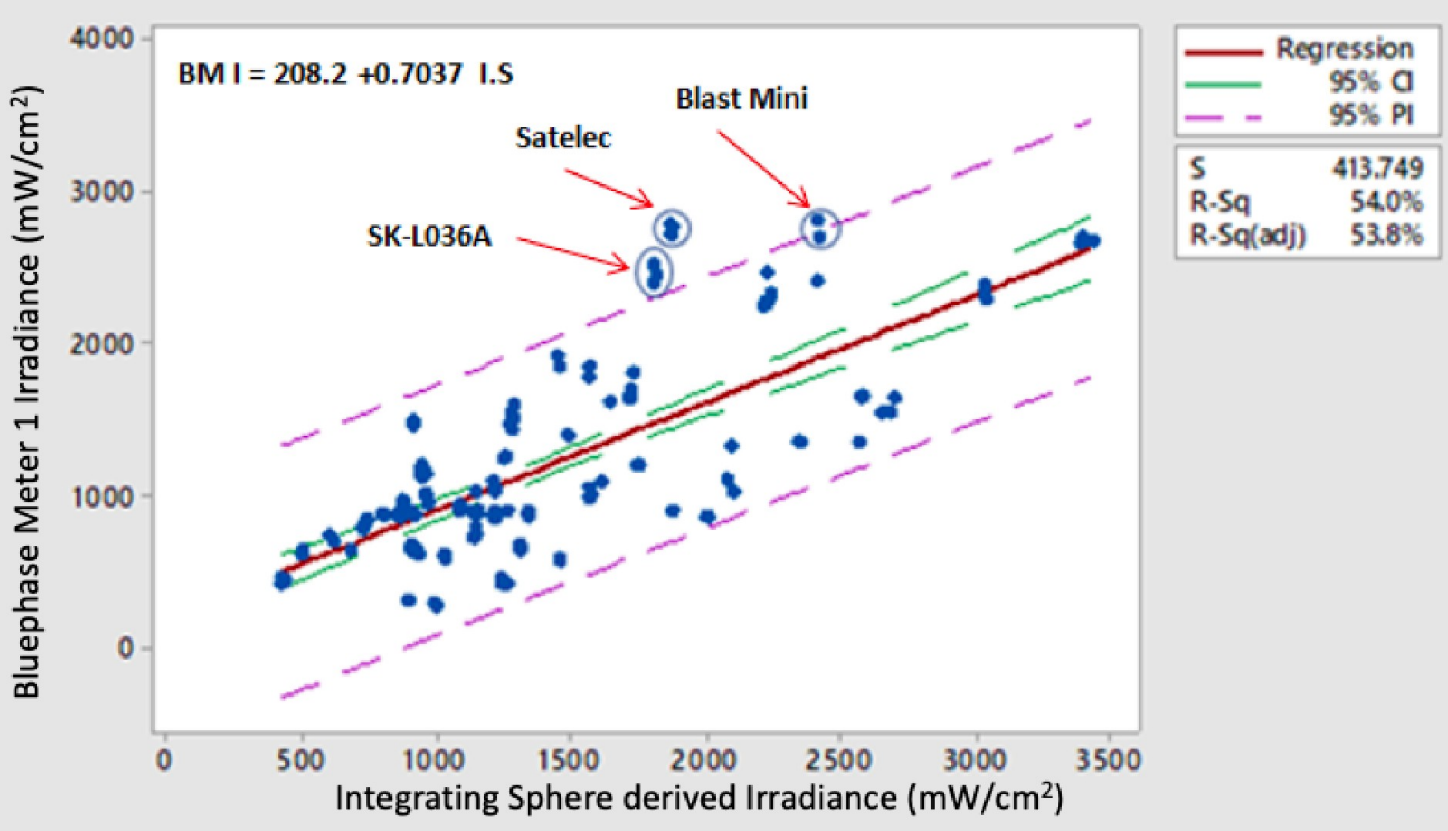
Fitted line plot of irradiance recorded with the BM I linear sensor radiometer versus integrating sphere derived irradiance for Type I and Type II LED-LCUs. High outliers (circled from left to right of the graph) were noted for the Spark SK-L036A, Satelec Supercharged, and Blast Mini LED-LCUs. A similar pattern was noted with the Ledex CM 4000 sensor. These two radiometers were the only ones with relatively narrow linear sensors. When beam profiles for these three units were inspected, it was noteworthy that all three units exhibited centrally located "hot spots" with low areas of light output on the outer regions of the light guide exit faces. Fig 7C and 7D show representative beam profiles for two of these three LCUs.

Using the MARC LC device, the irradiance from the central 4 mm diameter of the light guides of two LCUs (Elipar S10 and Elipar Deep Cure) was measured and compared with the mean irradiance over the full optically active area of the 10 mm diameter light guide. Hence, overall irradiance was greater for the Deep Cure unit (1303 mW/cm^2^) compared with the S10 (1149 mW/cm^2^). However, the irradiance over the central 4 mm diameter of the light guide output was greater for the S10 unit (1916 mW/cm^2^) than for the Deep Cure (1810 mW/cm^2^). The beam profile of the Deep Cure LCU revealed greater homogeneity compared with that of the S10 (Fig 7A & 7B). While these two LCUs had matching optically active diameters, their irradiance rankings reversed when they were measured using two different light collection areas. The beam profiles of two LCUs (Satelec Supercharged; Acteon and Spark SK-L036A) also revealed central areas of substantially higher irradiance compared with that towards the periphery of the light guide exit tip diameter (Fig 7C and 7D). High outliers on the fitted line plots for the two line-based sensor radiometers (BM I and Ledex CM 4000) were noted to correspond to these particular LCUs (Fig 8).

### 3.4 Light-curing unit tip diameter and optics

**Fig 9:**
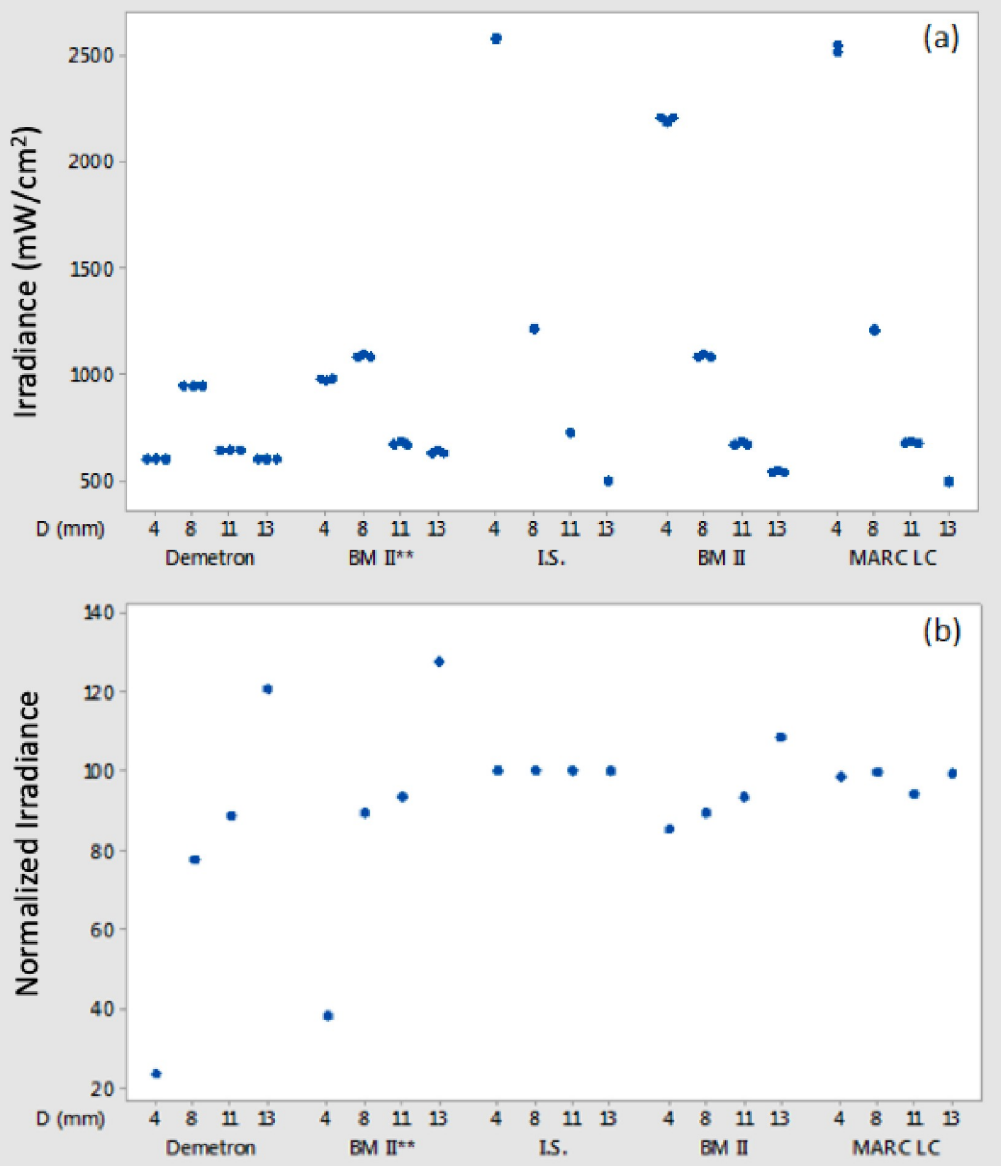
(a) Irradiance values calculated for the four tested guide diameters (D: 4, 8, 11, 13 mm) of the Demetron A2 LED LCU based on power (mW) readings recorded (1) using the integrating sphere fiber-coupled spectro-radiometer gold standard test method (2) the MARC LC instrument and irradiance values recorded using the Demetron L.E.D. radiometer and the BM II sn:1300001150. The BMII instructions for use state that irradiance values may be recorded for light guides between 6 mm and 12 mm diameter and power (mW) values for light guides between 5 mm and 13 mm diameter. A guide with mm increments between 6 mm and 12 mm is provided on the instrument base to allow guide tip diameter measurements for programming irradiance values from power (mW) measurements. The graph displays the raw data (**) irradiance values for this instrument, as recorded for the four light guides used with the Demetron A2 and the recalculated values for the 4 mm and 13 mm tips. The latter were based on the increase in area (from 4 mm to 6 mm diameter) or decrease in the area (from 13 mm to 12 mm) between the actual guide diameter tested and the programmed instrument software input based on using the nearest available guide diameter. Note how, when this is done, the BM II data approaches the GS results and how the MARC LC data is in close accord to the GS data set. The results confirm the value of using a meter light sensor area larger than any tested light source exit window area. (b) The same data as Fig 7A normalized relative to the mean integrating sphere (100%) gold standard data.

For the Demetron A2 LCU (4 mm tip), every DR underestimated the irradiance by 18 to 39% compared to the GS. Irradiance was determined with the BM II meters first by using the lowest programmable light guide diameter (6 mm) and then re-estimated according to a 2.25 times greater area correcting for tip diameter (Fig 9). When the S.P.E.C.3 LCU was tested in standard output mode, three radiometers gave significantly greater (P<0.05) irradiance readings with the 11 mm diameter tip while five gave significantly (P<0.05) greater readings with the 8 mm tip.

### 3.5 Influence of integration time on recorded power readings

**Fig 10:**
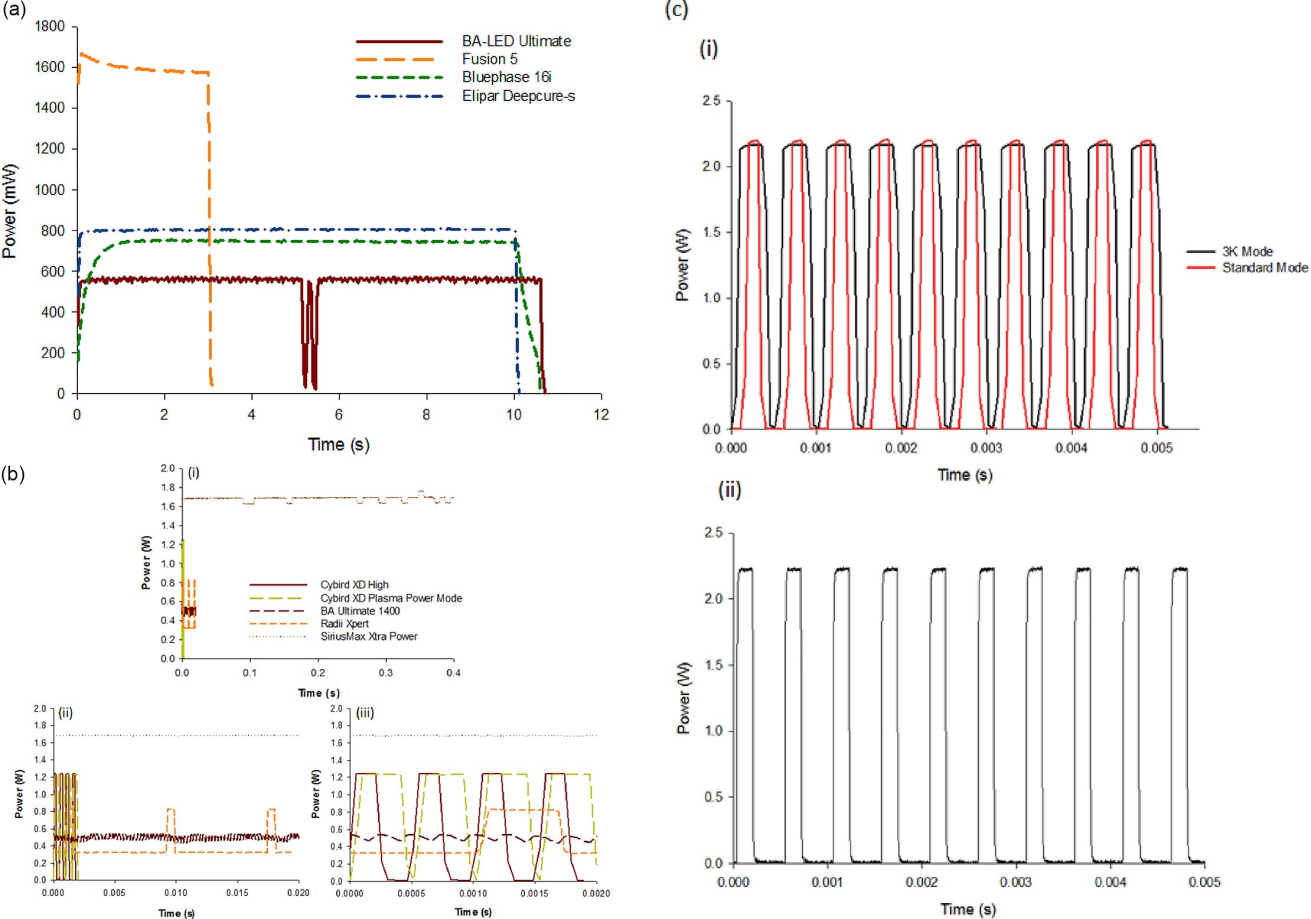
**(a)** Stable power output over time for the Elipar Deep Cure-S LED-LCU compared with other LCUs (BA Ultimate 1400, Bluephase 16i HIP mode, Fusion 5 Plasma power), recorded using the MARC LC spectrometer-based instrument acquiring data at 30 frames per second. A relatively slower rise in power output versus time is seen for the Bluephase 16i in "High Power" output mode while the output from the Fusion 5.0 declines slightly over the 3 s Plasma output mode run time. Note pulsing nature of light output for the BA Ultimate 1400 seen at the 30 Hz rate, (black) At ~5 s, the BA Ultimate 1400 LCU switches off briefly and immediately restarts. The pulsing output was found to relate to changes in the violet component of the light output over time. **(b)** Four of the eight LCUs from the 38 tested exhibiting pulsing output patterns in high speed (20 kHz) data acquisition mode with the PowerMax-Pro fast response power meter (S.P.E.C.3, Omega, I LED and Xlite 2 not shown). Fig 10Bi shows Power output (W) for the S.P.E.C.3 in standard and 3K modes. Ten pulses over 0.005s equalling a rate of 20 kHz for this LCU. Note similar peak power output in both modes with lower average power output for the standard mode due to longer off duty times between pulses. The manufacturers claimed irradiance values for these modes (1600 and 3000–3500 mW/cm^2^) corresponded closely with GS irradiance data recorded using the I.S./spectrometer test method (~1900 and ~3400 mW/cm^2^). Fig 10ii to iv show the outputs for the BA Ultimate 1400, Radii Xpert, in their standard operating modes. The Cybird XD LCU in High and Plasma power output modes and the Sirius Max in Xtra power mode. The standard output mode for the Sirius Max showed steady output with no pulsing behaviour. Fig 10ii to 10iii are the same apart from the different x-axis time scales. **(c) (i)** shows power output for the S.P.E.C.3 in standard and 3k modes. Ten pulses over 0.005s equalling a rate of 20 kHz for this LCU. Note similar peak power output in both modes with lower average power output for the standard mode due to longer off ‘duty’ times between pulses. Depending on tip diameter (8 or 11 mm), averaged power values recorded with GS method were ~1.45–1.55 W for 3K mode and 0.85–0.9 W for standard mode and ~0.1 W less as recorded with the LabMax-Pro as displayed here. The manufacturers claimed irradiance values for these modes (1600 and 3000–3500 mW/cm^2^) corresponded closely with GS irradiance data recorded using the I.S./spectrometer test method (~1900 and ~3400 mW/cm^2^). (ii) shows the standard output mode for the S.P.E.C.3 recorded this time with the LabMax-Pro operating in Snapshot mode at 625 kHz. Note changes in pulse shape between (i) and (ii) due to ~30-fold increase in acquisition rate.

Recording power output over the full exposure cycle time of each LCU with the MARC LC spectrometer-based instrument revealed additional information compared with the momentary or snapshot recordings of power captured by the BM II instruments, or in the way that the laboratory-grade power meters, and the GS integrating sphere were initially configured (Fig 10A). Power recorded with the high speed (20 kHz) acquisition mode of the PowerMax-Pro sensor revealed that eight of the LCUs under investigation (see Fig 10B for details of the names of the eight LCUs) had a high frequency pulsing power output in one or more modes which is not apparent at the normal 10 Hz acquisition rates commonly used with laboratory-grade power meters.

## 4. Discussion

Challenges exist when measuring the irradiance delivered by dental LED-LCUs [8]. The diversity of LCU types and irradiation protocols complicate the measurement of power output, spectral, and irradiance characteristics. Reporting of radiometric data requires appropriate nomenclature and precise physical definitions of key terms [8, 9]. Reference “GS" instruments for measuring radiant exitance have included scanning monochromators [10], laboratory-grade power meters [8, 14, 16, 17, 22] spectrometers, and integrating spheres [8, 9, 20, 21]. Commercial hand-held DRs differ in the way they convert light into electric current between instruments [17, 19]. Some have a small light-transmitting aperture window for taking peak irradiance measurements. In contrast, others have a large aperture for averaging the output from the entire light guide output [15].

Frequently, DRs incorporate bandpass filters to limit light transmission to specific wavelength ranges. *This may cause the spectral response of different meters to vary even though they share silicon photodiodes in common*. Furthermore, the vast majority of radiometers have smaller sensor areas than the tested areas of the light source exit windows, and therefore may not yield irradiance data in line with light source radiant exitance [22]. An exception is the large sensor area of the photovoltaic cell array used in the BM II, which will have contributed to its superior accuracy relative to the other tested radiometers.

*The supplementary investigation on a retrieved mirror attenuator of one of the BM II meters outlined in the second paragraph of section 3*.*2 of the results*, the BM II manufacturer likely uses an attenuator that exhibits higher violet wavelength transparency to compensate for the longer wavelength sensitivity of the photovoltaic cell array used in this DR (S3 Fig). Based on their findings, Kakeyama et al. [21] concluded that individual DRs had different wavelength sensitivities, which affected their accuracy. The researchers tested five radiometers against a laboratory-grade spectrometer control and concluded that the Cure Rite (Dentsply) radiometer was more sensitive to emissions in the 460–465 nm range and less so around 450 nm, but the converse for the BM I. As the Cure Rite was designed to test Halogen LCUs this result might be expected. The BM I and BM II radiometers were sensitive to all three waveband outputs of the AURA light source, whereas the Cure Rite radiometer was most sensitive at 470 nm (Table 4). Lee et al. [10] found that the irradiance ranking of five QTH-LCUs varied according to whether the light output was recorded between 400–520 nm or the narrower 410–500 nm wavelength range. In the current study, all DRs (except BM I and BM II) exhibited a greater response to wavelengths above 450 nm (P<0.05), which suggests that they are based on silicon photodiode sensors.

*The fact that the mean power readings for the Smartlite Focus tested with the BM II meters were 20% to 21% greater than for the corresponding Powermax-Pro power value* accords with the findings of the study by Shimokawa et al. [22]. It suggests that the manufacturer's claim of ± 10% accuracy for the instrument was based on Type I LED-LCUs. The BM II manufacturer does not market any Type II LCUs and recommends irradiance measurements be restricted to Type I, and only power measurements are taken for Type II LCUs. Manufacturers of DRs incorporate diffusing elements into their entrance apertures, but it has been shown that they do not completely account for regions of irradiance inhomogeneity from the LCU [22]. Other meters replace diffusers with light attenuating filters. The mirror attenuator light entrance “window” of the BM II has a highly reflective mirrored front surface. This may have been a factor in the higher mean power readings found for certain Type II lights compared to the integrating sphere control (S4 Fig). The LCUs in question had relatively well-collimated beams. Therefore, specular light reflectance from the mirrored surface may be redirected from the light source exit window back to the radiometer sensor, thereby increasing the measured irradiance. However, an integrating sphere only receives light directly from the source with no implication of reflected artifacts. Individual optics design may also influence light source exit and target reflectivity, which is the subject of further investigation.

Two DRs (Ledex CM 4000 and BM I) incorporate line sensors which are designed to measure effective light output diameter in order to be able to calculate irradiance. Shimokawa et al. [22] measured light-emitting (effective) tip diameters for their tested LCUs with a digital micrometer, with the BM II scale and also with the Ledex CM 4000-line sensor radiometer. None of the values matched, and as well as giving the greatest values, the Ledex radiometer was not able to rank the LCUs for irradiance correctly compared with a laboratory-grade power meter control. The line sensors of the Ledex and BM I radiometers (2 mm and 3 mm width, respectively) will overestimate irradiance readings of LCUs with central areas that have higher irradiance compared with the periphery of the exit window diameter. It is also important to note that the mismatch between the LCU emission area and the radiometer light collection area varied substantially between different LCU and radiometer combinations. Kakeyama et al. [21] suggested the need to develop a compact radiometer capable of being precisely calibrated, which can measure the output of any type of dental LCU accurately. Shimokawa et al. [22] endorsed this proposal and recommended that power output is likely to provide more useful data than irradiance, given the inherent inaccuracy of tip diameter measurements restricted to the nearest mm.

Modern dental LED-LCUs have very diverse design and output characteristics. Multi-spectral peak or so-called "polywave" units have been marketed to activate alternative photoinitiators that respond to violet light wavelengths (either for cosmetic purposes since camphorquinone may cause yellowing, or to increase polymerization rate since molar absorptivity of such alternatives is usually higher). Some LCUs vary their spectral output throughout the radiation cycle (BA Ultimate 1400, Bluephase G2 and 20i in soft start mode) and the Scanwave LCU [9, 23]. While light beams of broadband QTH sources show no evidence of spectral inhomogeneity, polywave dental LED units may have a spatially inhomogeneous spectral output [8, 24]. The effect of this inhomogeneity has been the subject of several studies, and there is debate about the influence of this on material properties [25–28]. Some authors report steep differences in material surface properties locally depending on light distribution [25–27]. In contrast, others cannot find any such effect [28]. Reasons for these contrasting results will likely lie in differences in exposure times, extent of cure, and material formulation differences as well as experimental design differences. Operator influence may be an even greater clinical variable.

Since modern LCUs are now used for a much broader range of clinical applications, many manufacturers have introduced additional light guide tips of various diameters and shapes for Type I LCUs. Previous studies have reported that commercial radiometers overestimate actual irradiance with larger diameter (10.5 to 12.5 mm) tips but underestimate irradiance values with smaller diameter (≤8.0 mm) guides [16, 17]. In the current work, when LCUs with different diameter guides were tested (Tables 1 and 2) with the 16 different models of radiometer (Table 3), even more varied test patterns were observed. In contrast to previous studies on DR response to LCUs with different tip diameters [16, 17], mixed results were again *found for the Demetron A2 and the S*.*P*.*E*.*C*.*3 LCU and for* the other three LCUs (Bluephase 20i and 16i and Demetron A2) tested with different tip diameters. The results confirm a previous study that concluded that only when radiometer readings were measured with the same standard light-sensing test aperture could results be correlated [15].

Using the BM II light tip diameter guide (Fig 6A) to measure the external diameter of light guides in 1 mm increments can impose a significant inaccuracy for Type I LCUs. For example, a difference of only 0.5 mm between the external 8.5 mm tip diameter (56.74mm^2^) and active 8.0 mm light-emitting diameter (50.26 mm^2^) can give a difference of 6.47 mm^2^ in the area and 12% difference in the irradiance. The optically active diameters for the Type I LCUs were, on average, 9% less than their external tip diameters and ranged from 1% (Prototype Scanwave / monofilament tip) to 19% lower (X-lite 3 and JAS-2001 B), and this explains why the agreement between the gold-standard IS and the BM II was lower for irradiance than for power data.

Power measurements taken with traditional thermopiles typically acquire data at 10 Hz and have a relatively slow response time [21]. Spectrometers are faster and can acquire data at rates typically up to 1 kHz. The fast response LabMax-Pro operates at 10 Hz in standard mode, 20 kHz in fast mode, and 625 kHz in Snapshot mode. As far as the authors are aware, this is the first time that high-speed acquisition behaviour of dental LCUs have been reported. Some LCUs exhibit the same peak power in 'High Power' and 'Plasma (or 3K)' modes with the only difference in the two output modes being the 'off' period between individual pulses (Fig 10B and 10C). The manufacturers’ stated peak irradiance values for the different tested modes of the Cybird (and S.P.E.C.3 LCUs) are based on averaged power measurements and not the peak pulse power outputs seen in Fig 10B and 10C. The pulsing output for these LCUs is presumably designed for thermal dissipation to prevent LED overheating and modulate the duty cycle, which may, therefore, regulate the radiant exposure. It is not apparent from manufacturers' LCU literature that a number of these lights pulse in their standard or supposed constant output modes.

DR measurements only provide an estimate of momentary light output and do not indicate the appropriate radiation time for a given material/LCU combination. Previous work has focussed on developing a practical test method to predict the setting time of any light-activated restorative material irrespective of light source or material formulation variables [29–32]. Any future reliable method would depend on refractive index change of the resin phase during cure relative to the fixed refractive index of the filler system as well as numerous other confounding test variables [6, 30, 32]. Light scattering through the test material is involved thus unfilled resins and translucent shade resin composites will show limited transmission changes during cure. There are differences in filler/resin refractive index between different manufacturers' materials. Developing such a test which could be readily performed in the dental office would eliminate the need for the dentist having to cross-reference between material instructions for use and a radiometer reading to determine an appropriate radiation time for a given thickness and shade of a particular material.

## 5. Final comments and possible directions for further development

A limitation of the current work is that in most cases only one example of each make and model of dental radiometer was tested. Current DRs cannot reliably estimate power or irradiance for a wide range of dental LED-LCUs. However, their value is that they may be used to periodically monitor the output of a LCU from new provided the test conditions are standardized (same DR, LCU operating mode and tip and etc.,) to guard against significant output decline. Manufacturers face major challenges in making an affordable and easy-to-use hand-held DR, which can accurately measure the power and/or irradiance of dental LCUs. Dentists will not be interested in purchasing laboratory-grade test instruments, which can prove to be very costly and more challenging to use. Even they have limitations such as the inability of most of them to register peak power of all LCUs unless operated at very high (20 KHz) data acquisition rates. It is believed that by addressing the issues with current DRs reported and discussed in the current investigation that dental manufacturers may be able to manufacture more reliable DRs based on innovative design features and sensor materials. Sensors which are larger than the light-emitting source area and have faster acquisition rates than current sensors, filters or attenuators which flatten the sensor spectral response, automated ways of measuring light source area and programs which allow power, as well as irradiance to be estimated, will be among the design features of new and improved DRs. New sensor materials, reductions in the cost of components, miniaturization of electronic components, and linking DR software to manufacturers' databases will all contribute to improvements in DR performance.

## 6. Conclusions

This study offers new insights into the possible reasons for DR inaccuracy and how they may possibly be addressed. Profound differences between actual and estimated irradiance were reported ranging from 7% to 535% of GS method for individual LCU/DR combinations–an order of magnitude greater than previously reported. Consequently, there is a need to use more reliable DRs for greater light measurement accuracy in a clinical setting. Despite this, the major value of currently available DRs lies in the fact that they may be used to periodically monitor the output of a LCU from new to guard against significant output decline in service.

Manufacturers should provide more accurate information on the output of their LCUs, including spectral emission characteristics and radiant power outputs of different modes over the full exposure period. Single irradiance values are of limited value and are often less reliable than the power measurements from which they are derived.

When researchers or manufacturers report irradiance values recorded using laboratory-grade test instruments, the light emitting diameter and area of the source, and test instrument sensor measurement diameter or area should always be stated along with the power.

Researchers who study resin photopolymerization should not rely on radiometers to assess the LCU output but rather report spectral radiant power output ideally over the full exposure cycle and emission spectrum characteristics. Current dental radiometers are not able to estimate these parameters.

## Supporting information

S1 Fig Radiant power for all tested Type I (a) and Type II (b) LCUs.
(DOCX)

S2 Fig **The calibration data of the AURA, Lumencor light source (light engine) with three spectral band outputs at λ**_**max**_ **= 405 nm, 470 nm, and 550 nm and Full Width Half Maximums (FWHM) of 14, 21 and 31 nm respectively was calibrated to deliver a pooled average of 1049 ± 146 mW/cm**^**2**^ **at each spectral band (left), and the spectral irradiance at each spectral band (middle).** It can be noted that the light source exhibits a homogenous flat-top beam profile at each spectral band (right) and therefore the effect of the smaller sensor size compared to the exit diameter of the light source is not likely to affect the calibration.
(DOCX)

S3 Fig **A supplementary investigation was conducted on a retrieved mirror attenuator from one of the BM II instruments (sn: 1300001134)’ light transmission was measured directly through a cosine corrector (top) and then with BM II mirrored attenuator interposed (bottom) using the 405 nm and 470 nm wavelengths of the Lumencor AURA Light Engine (set at** ~**1049 ± 146 mW/cm**^**2**^**).** ~40% higher transmission was found for the violet compared to the blue wavelength range testing with the mirrored attenuator explaining the flatter spectral response for this radiometer.
(DOCX)

S4 Fig (a) Specular reflection for the BM II mirrored attenuator and the light entrance diffuser of the Demetron LED radiometer or “Kerr Optics”. (b) Diffuse reflection (specular excluded) for the BM II and the Demetron “Kerr Optics” diffuser. Reflectance from the BM II is specular whereas it is diffuse from the “Kerr Optics” which is typical of the diffusers incorporated in nearly all dental radiometers. Note the reduced specular reflectance of the integrating sphere light for the BM II at shorter wavelengths consistent with the fact that this attenuator transmits a higher percentage of violet compared to blue compared to violet wavelengths. Spectra obtained using an Ocean Optics ISP-REF integrating sphere / spectrometer assembly. Reflectance waveband shown (380–550 nm) is the stated light sensing range for the BM II radiometer.
(DOCX)
