# Supplementary figures and images for: On the inaccuracies of dental radiometers
Source: PLoS One. 2021 Jan 29;16(1):e0245830. doi: 10.1371/journal.pone.0245830 (PMC7845964; doi:10.1371/journal.pone.0245830)

**S1 Fig:** Radiant power for all tested Type I (a) and Type II (b) LCUs

1. ***Type I***


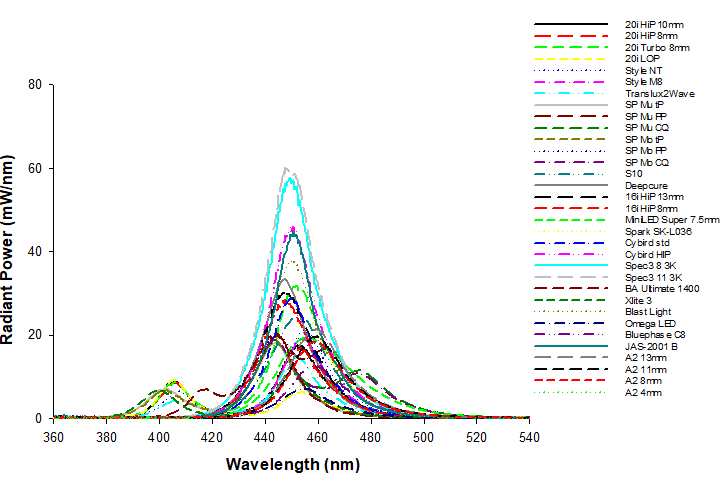


1. ***Type II***


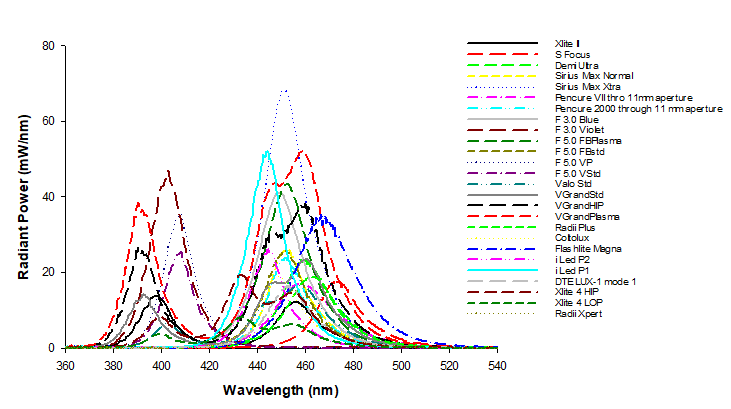

Supplement: S1 Fig — Radiant power for all tested Type I (a) and Type II (b) LCUs. (DOCX) [file pone.0245830.s001.docx]
