# Supplementary material for: On the inaccuracies of dental radiometers
Source: PLoS One. 2021 Jan 29;16(1):e0245830. doi: 10.1371/journal.pone.0245830 (PMC7845964; doi:10.1371/journal.pone.0245830)
Supplement: S2 Fig — The calibration data of the AURA, Lumencor light source (light engine) with three spectral band outputs at λmax = 405 nm, 470 nm, and 550 nm and Full Width Half Maximums (FWHM) of 14, 21 and 31 nm respectively was calibrated to deliver a pooled average of 1049 ± 146 mW/cm2 at each spectral band (left), and the spectral irradiance at each spectral band (middle). It can be noted that the light source exhibits a homogenous flat-top beam profile at each spectral band (right) and therefore the effect of the smaller sensor size compared to the exit diameter of the light source is not likely to affect the calibration. (DOCX) [file pone.0245830.s002.docx]

**S2 Fig:** The calibration data of the AURA, Lumencor light source (light engine) with three spectral band outputs at λ_max_ = 405 nm, 470 nm, and 550 nm and full width half maximums (FWHM) of 14, 21 and 31 nm respectively was calibrated to deliver a pooled average of 1049 ± 146 mW/cm^2^ at each spectral band (left), and the spectral irradiance at each spectral band (middle). It can be noted that the light source exhibits a homogenous flat-top beam profile at each spectral band (right) and therefore the effect of the smaller sensor size compared to the exit diameter of the light source is not likely to affect the calibration.


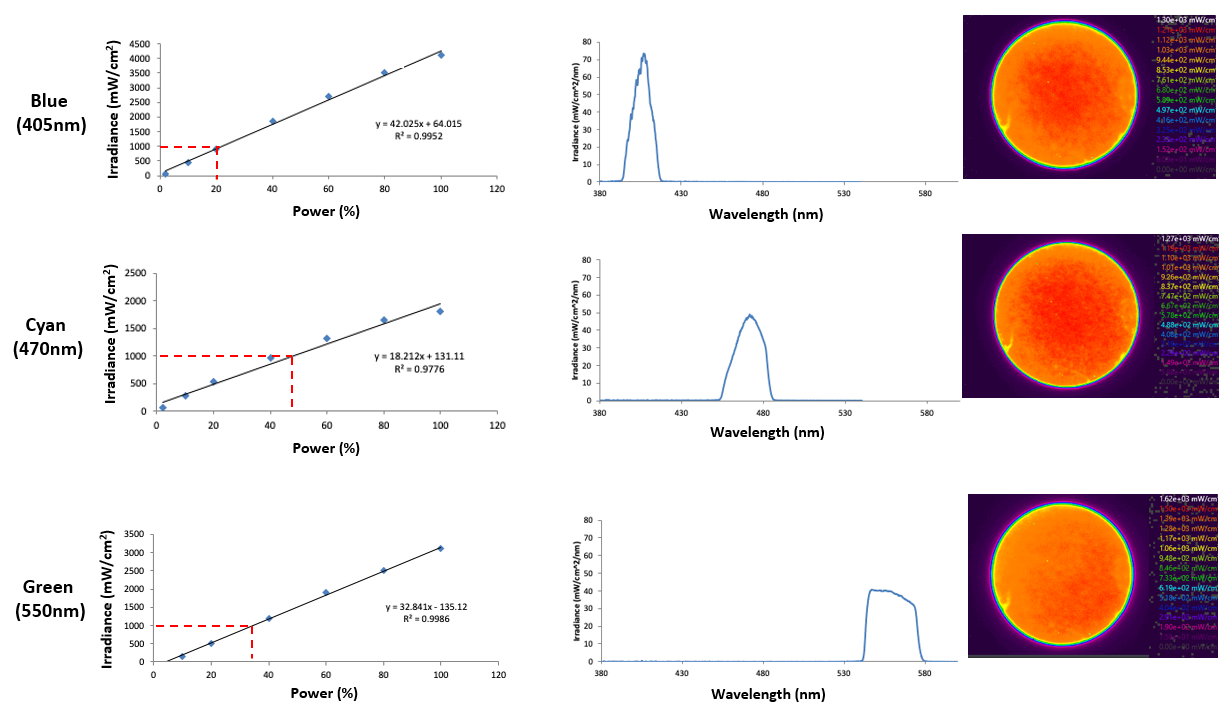


λ_max_ = 550nm

FWHM = 31nm

λ_max_ = 470nm

FWHM = 21nm

λ_max_ = 405nm

FWHM = 14nm
