# Supplementary material for: On the inaccuracies of dental radiometers
Source: PLoS One. 2021 Jan 29;16(1):e0245830. doi: 10.1371/journal.pone.0245830 (PMC7845964; doi:10.1371/journal.pone.0245830)

**S3 Fig**: A supplementary investigation was conducted on a retrieved mirror attenuator from one of the BM II instruments (sn: 1300001134)’ Light transmission was measured directly through a cosine corrector (top) and then with BM II mirrored attenuator interposed (bottom) using the 405 nm and 470 nm wavelengths of the Lumencor AURA Light Engine (set at ~1049 ± 146 mW/cm^2^). ~40% higher transmission was found for the violet compared to the blue wavelength range testing with the mirrored attenuator explaining the flatter spectral response for this radiometer.


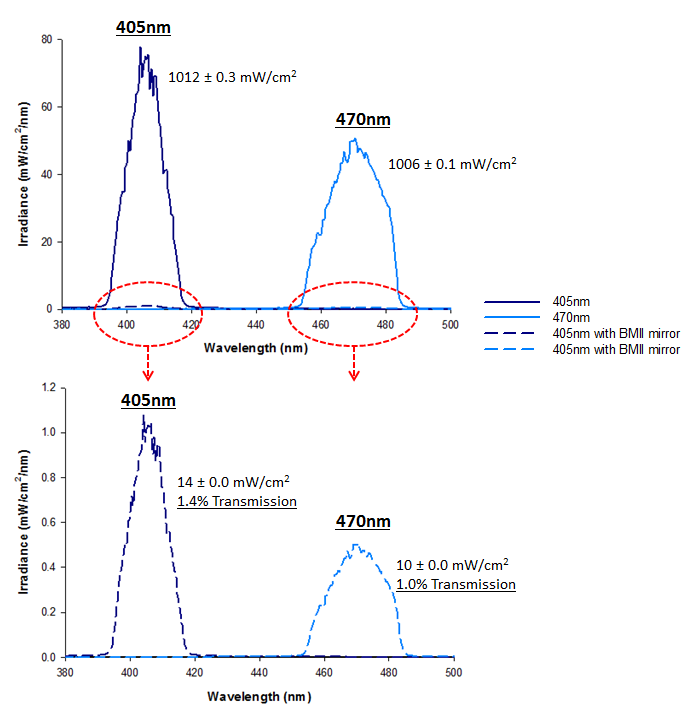

Supplement: S3 Fig — A supplementary investigation was conducted on a retrieved mirror attenuator from one of the BM II instruments (sn: 1300001134)’ light transmission was measured directly through a cosine corrector (top) and then with BM II mirrored attenuator interposed (bottom) using the 405 nm and 470 nm wavelengths of the Lumencor AURA Light Engine (set at ~1049 ± 146 mW/cm2). ~40% higher transmission was found for the violet compared to the blue wavelength range testing with the mirrored attenuator explaining the flatter spectral response for this radiometer. (DOCX) [file pone.0245830.s003.docx]
