# Supplementary material for: On the inaccuracies of dental radiometers
Source: PLoS One. 2021 Jan 29;16(1):e0245830. doi: 10.1371/journal.pone.0245830 (PMC7845964; doi:10.1371/journal.pone.0245830)

**S4 Fig:** (a) Specular reflection for the BM II mirrored attenuator and the light entrance diffuser of the Demetron LED radiometer or “Kerr Optics”. (b) Diffuse reflection (specular excluded) for the BM II and the Demetron “Kerr Optics” diffuser. Reflectance from the BM II is specular whereas it is diffuse from the “Kerr Optics” which is typical of the diffusers incorporated in nearly all dental radiometers. Note the reduced specular reflectance of the integrating sphere light for the BM II at shorter wavelengths consistent with the fact that this attenuator transmits a higher percentage of violet compared to blue compared to violet wavelengths. Spectra obtained using an Ocean Optics ISP-REF integrating sphere / spectrometer assembly. Reflectance waveband shown (380 – 550 nm) is the stated light sensing range for the BM II radiometer.

(a)


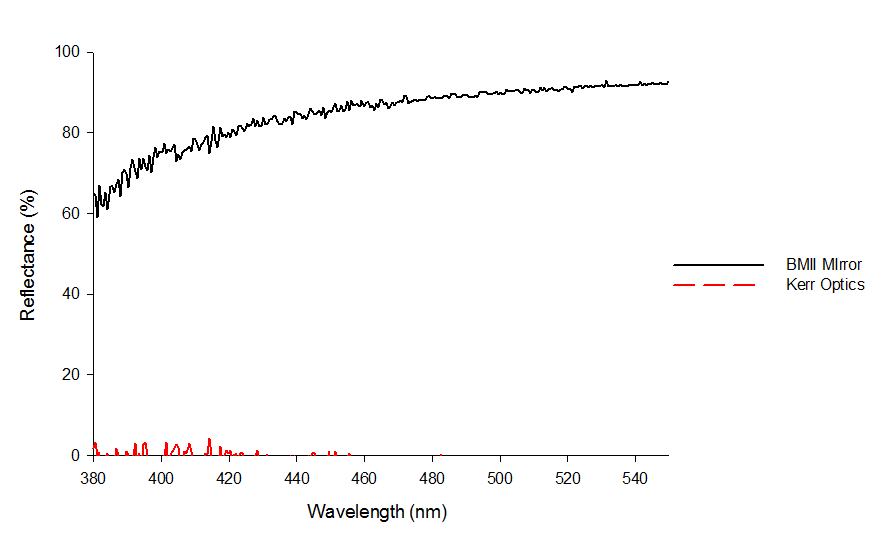


(b)


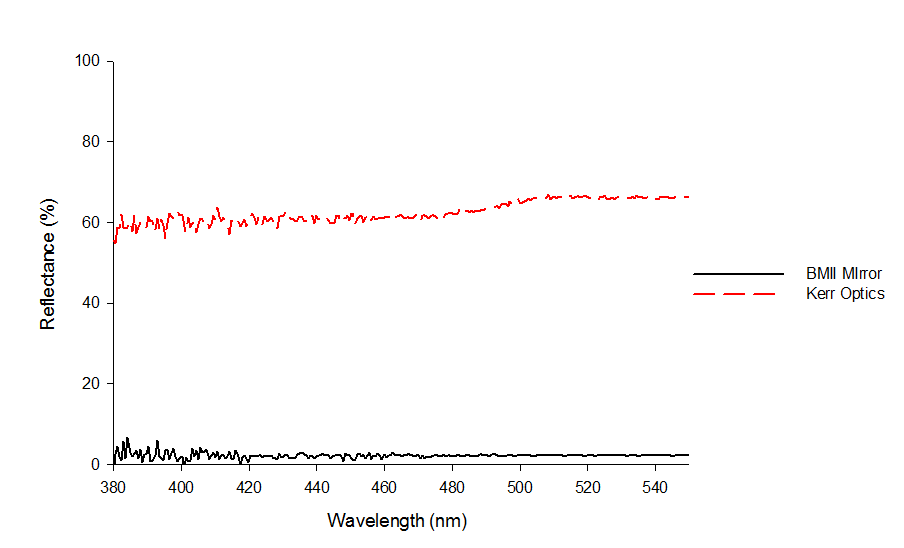

Supplement: S4 Fig — (a) Specular reflection for the BM II mirrored attenuator and the light entrance diffuser of the Demetron LED radiometer or “Kerr Optics”. (b) Diffuse reflection (specular excluded) for the BM II and the Demetron “Kerr Optics” diffuser. Reflectance from the BM II is specular whereas it is diffuse from the “Kerr Optics” which is typical of the diffusers incorporated in nearly all dental radiometers. Note the reduced specular reflectance of the integrating sphere light for the BM II at shorter wavelengths consistent with the fact that this attenuator transmits a higher percentage of violet compared to blue compared to violet wavelengths. Spectra obtained using an Ocean Optics ISP-REF integrating sphere / spectrometer assembly. Reflectance waveband shown (380–550 nm) is the stated light sensing range for the BM II radiometer. (DOCX) [file pone.0245830.s004.docx]
